# Neuronal aging causes mislocalization of splicing proteins and unchecked cellular stress

**DOI:** 10.1038/s41593-025-01952-z

**Published:** 2025-06-02

**Authors:** Kevin Rhine, Rachel Li, Hema M. Kopalle, Katherine Rothamel, Xuezhen Ge, Elle Epstein, Orel Mizrahi, Assael A. Madrigal, Hsuan-Lin Her, Trent A. Gomberg, Anita Hermann, Joshua L. Schwartz, Amanda J. Daniels, Uri Manor, John Ravits, Robert A. J. Signer, Eric J. Bennett, Gene W. Yeo

**Affiliations:** 1 Department of Cellular & Molecular Medicine, University of California San Diego, La Jolla, CA USA; 2 Sanford Stem Cell Institute Innovation Center and Sanford Consortium for Regenerative Medicine, University of California San Diego, La Jolla, CA USA; 3 Institute for Genomic Medicine, University of California San Diego, La Jolla, CA USA; 4 Waitt Advanced Biophotonics Center, Salk Institute for Biological Studies, La Jolla, CA USA; 5 Biomedical Sciences Graduate Program, University of California San Diego, La Jolla, CA USA; 6 Biological Sciences Graduate Program, University of California San Diego, La Jolla, CA USA; 7 Center for RNA Technologies & Therapeutics, University of California San Diego, La Jolla, CA USA; 8 Department of Cell & Developmental Biology, University of California San Diego, La Jolla, CA USA; 9 Bioinformatics & Systems Biology Graduate Program, University of California San Diego, La Jolla, CA USA; 10 Department of Neurosciences, School of Medicine, University of California San Diego, La Jolla, CA USA; 11 Division of Regenerative Medicine, Department of Medicine, Sanford Stem Cell Institute, Moores Cancer Center, University of California San Diego, La Jolla, CA USA

**Keywords:** Neural ageing, Amyotrophic lateral sclerosis, Cellular neuroscience, Alternative splicing, Mechanisms of disease

## Abstract

Aging is one of the most prominent risk factors for neurodegeneration, yet the molecular mechanisms underlying the deterioration of old neurons are mostly unknown. To efficiently study neurodegeneration in the context of aging, we transdifferentiated primary human fibroblasts from aged healthy donors directly into neurons, which retained their aging hallmarks, and we verified key findings in aged human and mouse brain tissue. Here we show that aged neurons are broadly depleted of RNA-binding proteins, especially spliceosome components. Intriguingly, splicing proteins—like the dementia- and ALS-associated protein TDP-43—mislocalize to the cytoplasm in aged neurons, which leads to widespread alternative splicing. Cytoplasmic spliceosome components are typically recruited to stress granules, but aged neurons suffer from chronic cellular stress that prevents this sequestration. We link chronic stress to the malfunctioning ubiquitylation machinery, poor HSP90α chaperone activity and the failure to respond to new stress events. Together, our data demonstrate that aging-linked deterioration of RNA biology is a key driver of poor resiliency in aged neurons.

## Abstract (web summary)

Rhine et al. find that neuronal aging leads to widespread dysregulation of RNA biology, including mislocalization of splicing proteins like TDP-43, chronic cellular stress and reduced resiliency.

## Main

Our rapidly aging population poses one of the largest public health challenges in a generation^1^. A substantial portion of the public health burden is driven by neurodegenerative diseases like dementia, Parkinson’s disease and amyotrophic lateral sclerosis (ALS), which all increase in incidence rate as a function of age^2^. These pathologies are collectively defined by declining neuronal health, which manifests in devastating symptoms that are costly to manage and greatly reduce the overall quality of life in the aged population^3^. Systematic efforts over the past decades have identified biomarkers that are linked to neurodegeneration, but the vast majority of neurodegeneration cases have no familial history or single causal mutation^4^. Even in mutation-driven neurodegenerative pathologies, onset of symptoms is typically later in life, indicating that aging is an important contributor to disease. Therefore, it is of critical importance to understand how aging alone alters the molecular landscape of neurons and predisposes neurons for poor resiliency to external and internal stress.

Aging is generally linked to several molecular processes^5^. In neurons, the accumulation of aggregated proteins, dysregulation of oxidative phosphorylation and mislocalization of disease-associated proteins are impacted by age and neurodegeneration-linked mutations^6–8^. However, the molecular interplay between these diverse cellular functions, their relative contribution to neurodegeneration and the importance of other implicated pathways are unknown, especially in an aged neuron that is susceptible to neurodegeneration. For example, the RNA-binding protein (RBP) TDP-43 forms cytoplasmic aggregates in >95% of patients with ALS, and it directs the proper splicing of neuronal cytoskeletal proteins^9,10^. Yet we know little about how TDP-43 is initially mislocalized in patients with ALS, nor how it eventually forms aggregates in the absence of an ALS-linked mutation^11^. More broadly, RBPs and RNA biology are disrupted in neurodegeneration^12,13^, but it is unclear how the natural aging of neurons contributes to this dysregulation.

Here we leverage transdifferentiation to study the RNA biology of neurons in the context of aging. Transdifferentiation is an efficient means to study aging: human primary fibroblasts are directly converted to neurons, bypassing the pluripotent stem cell state that reverses most aging-associated damage^14–16^. In transdifferentiated neurons, we show that RNA metabolism is broadly dysregulated—key splicing proteins are mislocalized, the RBP-mediated stress response is chronically active and overall resilience to further acute stress is dampened. Depletion of the chaperone HSP90α from stress granules leads to their chronic retention. Notably, the aging human brain also displays the hallmarks of RBP dysregulation and chronic stress. Our findings demonstrate how aging alone functionally destabilizes the neuronal stress response and TDP-43-mediated splicing, which bridges a substantial gap in our understanding of how aging predisposes neurons toward neurodegeneration.

## Results

### RBPs are downregulated in aged neurons

**Fig. 1:**
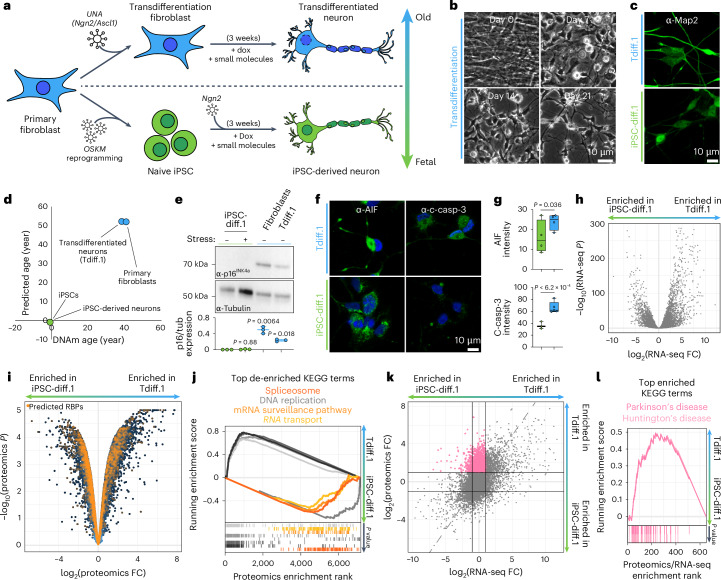
Aging leads to depletion of RBPs. **a**, Schematic representation of the transdifferentiation approach. **b**, Brightfield images of fibroblasts undergoing transdifferentiation. Scale bar = 10 μm. **c**, Confocal fluorescence images of transdifferentiated (Tdiff) and iPSC-derived (iPSC-diff) neurons stained for the neuronal marker Map2. Scale bar = 10 μm. **d**, Scatter plot of the predicted age and CpG methylation (DNAm) age of the indicated cell types. **e**, Top, representative western blot of p16^INK4A^ and tubulin expression in the indicated cell types. Bottom, quantification of average p16^INK4A^-to-tubulin expression for *n* = 3 blots, where the centerline is the median and the error bars denote the range of data values; statistics were calculated using a two-tailed Welch’s *t* test compared to the unstressed iPSC-derived sample. **f**, Confocal fluorescence images of the indicated cell types stained for c-casp-3 or AIF (green) with DAPI (blue). Scale bar = 10 μm. **g**, Quantification of the average fluorescence intensity of the c-casp-3 (top) or AIF (bottom) channels from **f** for iPSC-diff (green) and Tdiff (blue) neurons (*n* = 3 replicates), where the centerline is the median, the box bounds encompass the 25th–75th percentile values and error bars denote the range of data values; statistics were calculated using a two-tailed Welch’s *t* test. Error bars denote s.d. **h**, Volcano plot of differential expression of all detected transcripts as determined by RNA-seq in transdifferentiated and iPSC-derived neurons (*n* = 3 replicates); *P* values were calculated using a two-tailed Welch’s *t* test. **i**, Same as **h**, but for whole-cell MS (*n* = 3 replicates); orange points denote predicted RBPs^62^. **j**, Running enrichment score plot of the top eight most significant KEGG terms for the proteomics data in **i**; RNA metabolic pathways are highlighted in shades of orange. **k**, Scatter plot of RNA-seq and proteomic differential expression for all detected transcripts. All proteins that were significantly enriched in Tdiff neurons but had normal transcript levels are highlighted pink. **l**, Running enrichment score of the most significant KEGG terms for the high proteomics/normal transcript (pink) region in **k**. c-casp-3, cleaved caspase-3; FC, fold change. Source data

**Extended Data Fig. 1:**
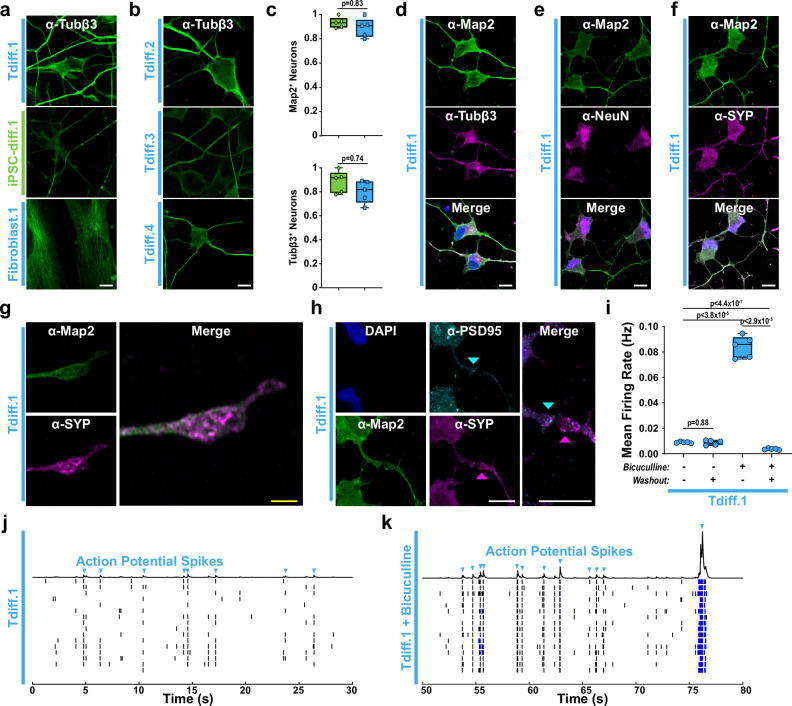
Transdifferentiation results in functionally mature neurons. **a**, Confocal fluorescence images of isogenic transdifferentiated neurons (Tdiff.1), iPSC-derived neurons (iPSC-diff.1) and fibroblasts (fibroblast.1) stained for tubulin-β3 (Tubβ3). Scale bar = 10 μm. **b**, Confocal fluorescence images of transdifferentiated neuronal lines (Tdiff.2–4) stained for Tubβ3. Scale bar = 10 μm. **c**, Quantification of the fraction of Map2^+^ and Tubβ3^+^ neurons (n = 5 replicates) in the iPSC-diff.1 (green) or Tdiff.1 (blue) lines. The box plot represents the 25th to 75th percentile of data values, the centerline is the median and the error bars denote the range of all values. Statistics were calculated using a two-tailed Welch’s. **d**, Confocal Immunofluorescence images of co-stained Map2 and Tubβ3 in Tdiff.1 neurons. Scale bar = 10 μm. **e**, Same as **d**, but for Map2 and NeuN/RBFOX3. **f**, Same as **d**, but for Map2 and synaptophysin (SYP). **g**, Airyscan immunofluorescence images of co-stained Map2 and synaptophysin in the neurites of Tdiff.1 neurons. Yellow scale bar = 2 μm. **h**, Confocal immunofluorescence images of co-stained DAPI (blue), Map2 (green), PSD95 (cyan) and synaptophysin (magenta) in Tdiff.1 neurons. The cyan arrow indicates the postsynaptic neuron, whereas the magenta arrow indicates the presynaptic neuron. Scale bar = 10 μm. **i**, Quantification of the average firing rate detected by MEA for Tdiff.1 neurons (n = 5 replicates) with and without bicuculline treatment before and after washout. The box plot represents the 25th to 75th percentile of data values, the centerline is the median and the error bars denote the range of all values. Statistics were calculated using a two-tailed Welch’s t test. **j**, Example of action potential spikes in untreated Tdiff.1 neurons. **k**, Same as **j**, but for Tdiff.1 neurons treated with bicuculline. Dark blue bars represent ‘bursting’.

We generated isogenic transdifferentiated (Tdiff.1) and induced pluripotent stem cell (iPSC)-derived neurons (iPSC-diff.1) by transducing wild-type patient-derived fibroblasts or iPSCs, respectively, with lentivirus encoding doxycycline-inducible neuronal transcription factors (Fig. 1a). After differentiating in the presence of doxycycline and other small molecules for 3 weeks, we successfully produced >90% Map2^+^ and >80% Tubβ3^+^ neurons from fibroblasts and iPSCs (Fig. 1b,c and Extended Data Fig. 1a–d), and the transdifferentiated neurons expressed several neuron-specific markers, including NeuN/RBFOX3 in the nucleus and synaptophysin at neurites (Extended Data Fig. 1e–g). In addition, mature synaptic connections were observed (Extended Data Fig. 1h). Transdifferentiated neurons also fired both spontaneous and induced action potentials as measured by a multielectrode array (MEA; Extended Data Fig. 1i–k). We then verified that transdifferentiated neurons retained key aging markers. Bisulfite sequencing of CpG methylation, which can be used to estimate biological age^17^, indicated that the transdifferentiated neurons were similar in age to the original donor fibroblasts, whereas the iPSC-derived neurons and iPSCs exhibited fetal-like methylation patterns (Fig. 1d). Likewise, transdifferentiated neurons expressed higher levels of the senescence marker p16^INK4A^ (Fig. 1e) and the apoptotic markers pro-caspase-3 and AIF (Fig. 1f,g), all of which are associated with aging^18–20^. Therefore, we used transdifferentiation to study neuronal aging.

**Extended Data Fig. 2:**
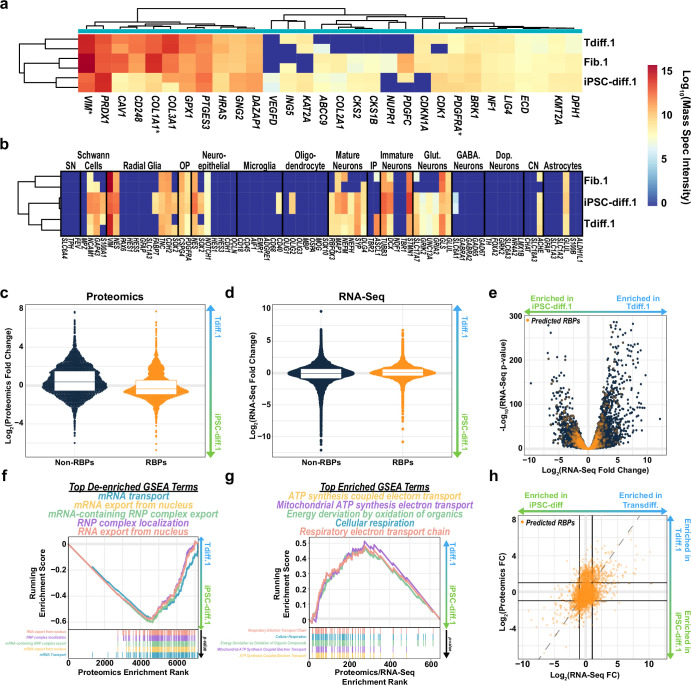
RNA metabolism pathways are highly depleted from transdifferentiated neurons. **a**, Heatmap of log_10_(mass spectrometry intensity) of all detected proteins from the gene ontology term fibroblast proliferation (GO:0048144) in the indicated cell types (n = 2 replicates). Well-known fibroblast markers were also manually added (indicated with an asterisk next to the protein name). **b**, Same as **a**, but for neuronal markers. SN, serotonergic neurons; OP, oligodendrocyte precursor; IP, intermediate progenitor; Glut. neurons, glutamatergic neurons; Dop. neurons, dopaminergic neurons; CN, cholinergic neurons. **c**, Distribution of the log_2_(proteomics fold change) of all detected non-RBPs (navy blue) and RBPs (orange; n = 3 replicates). The line within the box plot represents the median for the indicated population, and the box bounds represent the 25th to 75th percentile of data. **d**, Same as **c**, but for the RNA-seq data (n = 3 replicates). **e**, Volcano plot of RNA-seq data (n = 3 replicates) from Fig. 1h but with predicted RBPs highlighted in orange. P values were calculated using a two-tailed Welch’s t test. **f**, Plot of the running enrichment score of the top GSEA terms for the proteomics data. **g**, Plot of the running enrichment score of the top GSEA terms for the proteins with high expression but normal transcript levels (the pink region in Fig. 1h). **h**, Scatter plot of proteomics versus RNA-seq enrichment as in Fig. 1k (n = 3 replicates) but with only predicted RBPs plotted in orange.

RNA sequencing (RNA-seq) and differential expression analysis of the isogenic transdifferentiated and iPSC-derived neurons revealed that over 4,000 transcripts were significantly differentially expressed in transdifferentiated neurons (Fig. 1h). The upregulated pathways included the immune response and the lysosome, which are commonly upregulated in aged cells and further confirmed that transdifferentiated neurons retained expression of aging-linked genes^21^ (Supplementary Table 1). Whole-cell quantitative proteomics confirmed that most fibroblast markers were expressed at similar levels in iPSC-derived and transdifferentiated neurons, whereas mature and glutamatergic neuronal markers were highly upregulated in both cell types (Extended Data Fig. 2a,b), again indicating that our transdifferentiation process successfully converted fibroblasts into neurons. Notably, our proteomics data also revealed that known RBPs were broadly depleted in transdifferentiated neurons despite unchanged RBP transcript levels (Fig. 1i and Extended Data Fig. 2c–e). Consistent with this depletion, the most de-enriched metabolic pathways of transdifferentiated neurons involved RNA metabolic processes like the spliceosome, the mRNA surveillance pathway and RNA transport (Fig. 1j and Supplementary Table 2). Gene set enrichment analysis likewise showed depletion of RBP-associated pathways in transdifferentiated neurons relative to iPSC-derived neurons (Extended Data Fig. 2f). By mapping the proteomics data to the associated transcript levels, we also identified a large population of genes that were expressed highly as proteins but had normal or depleted transcript levels in transdifferentiated neurons (Fig. 1k). These highly retained proteins were associated with neurodegenerative pathways and oxidative phosphorylation (Fig. 1l and Extended Data Fig. 2g). Given that oxidative phosphorylation was also upregulated more broadly in our proteomics data (Fig. 1j), we hypothesized that aged neurons were prioritizing metabolic pathways over RNA-related processes (Extended Data Fig. 2h), which may lead to destabilization of RNA biology.

### Spliceosome proteins are mislocalized in aged neurons

**Fig. 2:**
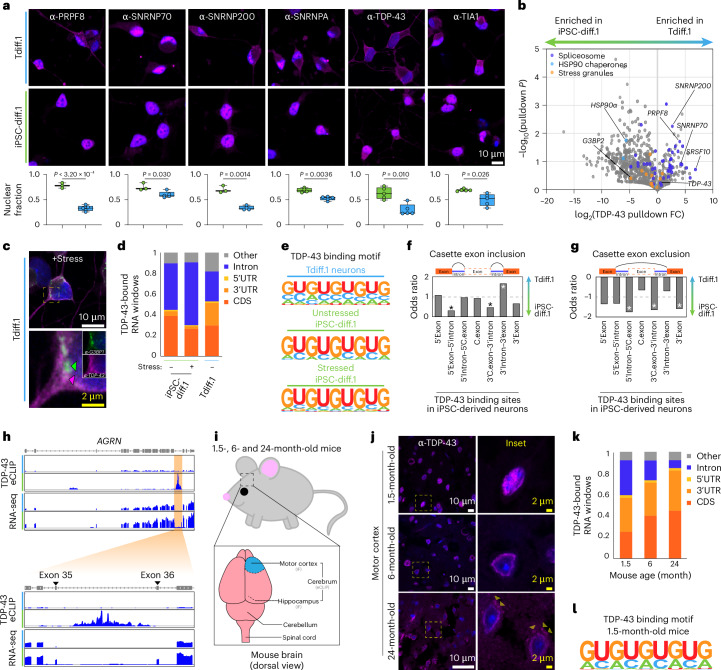
Splicing is dysregulated in aged neurons due to mislocalization of spliceosome components. **a**, Confocal fluorescence images of transdifferentiated (top row, blue) and iPSC-derived (middle row, green) neurons stained for the indicated spliceosome proteins in magenta with DAPI in blue; the average fraction of nuclear protein was calculated for each cell type (*n* = 3 replicates) and is plotted (bottom row) where the centerline denotes the median, the box bounds encompass the 25th–75th percentile values and the error bars denote the range of data values. Statistics were calculated using a two-tailed Welch’s *t* test. Scale bar = 10 μm. **b**, Volcano plot of AP-MS of TDP-43 (*n* = 2 replicates) in the indicated neurons (*n* = 2 replicates). Detected proteins in the indicated KEGG terms are highlighted. The *P* value was calculated using a two-tailed Welch’s *t* test. **c**, Airyscan fluorescence images of transdifferentiated neurons treated with sodium arsenite for 1 h and stained for G3BP1 and TDP-43. White scale bar = 10 μm; yellow scale bar = 2 μm. **d**, Fraction of TDP-43-bound RNA windows detected by eCLIP (*n* = 2 replicates) within each of the indicated transcript elements for transdifferentiated and iPSC-derived neurons. **e**, The top RNA-binding motif for TDP-43 eCLIP in the indicated cell types/conditions (*n* = 2 replicates). **f**, Exon inclusion odds ratio for transcripts detected by RNA-seq (*n* = 3 replicates) at the indicated loci around each exon with a TDP-43 binding site in iPSC-derived neurons, where *P < 0.05 calculated by a likelihood-ratio test. **g**, Same as **f**, but for exon exclusion. **h**, TDP-43 binding and RNA-seq intensity tracks for the *AGRN* locus in the indicated cell types. The orange highlighted region is depicted in the lower panel. **i**, Schematic representation of the mouse brain used for IF and eCLIP. **j**, Confocal fluorescence images of TDP-43 (magenta) and DAPI (blue) in the motor cortex of 1.5-, 6- and 24-month-old mice; the yellow dashed box indicates the inset region. White scale bar = 10 μm; yellow scale bar = 2 μm. **k**, Same as **d**, but for the indicated mouse ages (*n* = 2 mice per cohort). **l**, Same as **e**, but for the 1.5-month-old mouse brain. IF, immunofluorescence.

**Extended Data Fig. 3:**
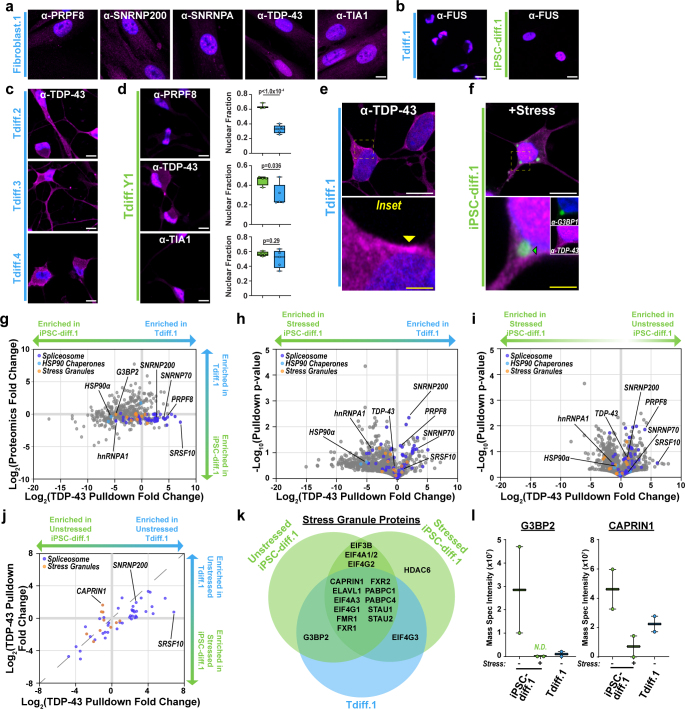
TDP-43 does not interact with stress granule proteins in transdifferentiated neurons. **a**–**c**, Confocal fluorescence images of the splicing proteins in the indicated cell types. Scale bar = 10 μm. **d**, Left, confocal fluorescence images of the indicated splicing proteins in the Tdiff.Y1 neuron line; Scale bar = 10 μm. Right, quantification of the nuclear fraction (n = 3 replicates) of TdiffY.1 versus Tdiff.1 (reproduced from Fig. 2a) for the indicated spliceosome protein. The box plot represents the 25th to 75th percentile of all values, the centerline denotes the median and the error bars represent the range of all values. Statistics were calculated using a two-tailed Welch’s t test. **e**, Airyscan fluorescence images of TDP-43 in transdifferentiated neurons. White scale bar = 10 μm; yellow scale bar = 2 μm. **f**, Airyscan fluorescence images of TDP-43 and G3BP1 in iPSC-derived neurons treated with arsenite for 1 h. White scale bar = 10 μm; yellow scale bar = 2 μm. **g**, Scatter plot of TDP-43 pulldown fold change plotted with the proteomic fold change for each detected protein (n = 2 replicates). P values were detected using a two-tailed Welch’s t test. **h**, Volcano plot of all detected proteins identified by TDP-43 pulldown and mass spectroscopy for Tdiff.1 neurons versus iPSC-diff.1 neurons (n = 2 replicates) treated with sodium arsenite for 1 h. P values were calculated using a two-tailed Welch’s t test. Detected proteins were highlighted as in **g**. **i**, Same as **h**, but for unstressed and stressed iPSC-diff.1 neurons. **j**, Scatter plot of the TDP-43 pulldown fold change for spliceosome (dark blue) and stress granule (orange) proteins in Tdiff.1 neurons compared to unstressed and stressed iPSC-diff.1 neurons (n = 2 replicates). **k**, Venn diagram of which stress granule proteins were detected in any replicate of the indicated neuron TDP-43 pulldown (n = 2 replicates). **l**, Raw mass spectrometry values of two core stress granules (G3BP2 and CAPRIN1) detected by TDP-43 pulldown.

Because the spliceosome was the most de-enriched of the RBP metabolism terms in transdifferentiated neurons (Fig. 1j), we performed immunofluorescence experiments to examine the subcellular localization of splicing proteins. Strikingly, we found that several spliceosome components were significantly mislocalized from the nucleus to the cytoplasm in transdifferentiated neurons compared to the isogenic iPSC-derived neurons (Fig. 2a). The mislocalization spanned across the various functional components of the spliceosome, including the U1 complex (SNRNP70 and SNRNPA), the tri-SNRNP complex (PRPF8 and SNRNP200) and proteins that direct alternative splicing (TDP-43 and TIA1)^10,22,23^. Notably, aged fibroblasts displayed a lower degree of splicing protein mislocalization (Extended Data Fig. 3a). Other proteins that can mediate alternative splicing, like FUS, were not substantially depleted from the nucleus (Extended Data Fig. 3a,b), perhaps because FUS has another nuclear role in the DNA damage response^24^. Mislocalization of TDP-43 was observed in multiple transdifferentiated neuronal lines (Extended Data Fig. 3c), but not in neurons that were transdifferentiated from young neonatal fibroblasts (Extended Data Fig. 3d).

TDP-43 aggregation is implicated in >95% of ALS cases and >50% of Alzheimer’s cases. In iPSC-derived neurons, prolonged stress treatments^25^ or truncation of the TDP-43 nuclear localization signal^26^ is required to observe TDP-43 mislocalization and aggregation. In transdifferentiated neurons, super-resolution microscopy confirmed basal TDP-43 mislocalization and further revealed TDP-43 foci in the soma of these neurons without any exogenous manipulation (Extended Data Fig. 3e). Treatment with sodium arsenite, an acute oxidative stressor, for 1 h further showed that TDP-43 foci in the neuronal soma were distinct from canonical stress granules, which typically integrate TDP-43 but instead clustered independently in transdifferentiated neurons (Fig. 2b,c and Extended Data Fig. 3f). Interrogation of TDP-43 interactions by affinity-pulldown mass spectrometry (AP-MS) from iPSC-derived and transdifferentiated neurons revealed that spliceosome proteins were highly enriched in the TDP-43 interactome of transdifferentiated neurons despite their lower protein expression (Fig. 2b, Extended Data Fig. 3g and Supplementary Table 3). Meanwhile, stress granule components like G3BP2 and Caprin1 were de-enriched in transdifferentiated neurons even when compared to TDP-43 pulldown in stressed iPSC-derived neurons (Fig. 2b and Extended Data Fig. 3h–l). The strong association between TDP-43 and spliceosome components in aged neurons could not be recapitulated by simply treating iPSC-derived neurons with sodium arsenite (Extended Data Fig. 3h–j), indicating that it is an aging-specific phenomenon.

**Extended Data Fig. 4:**
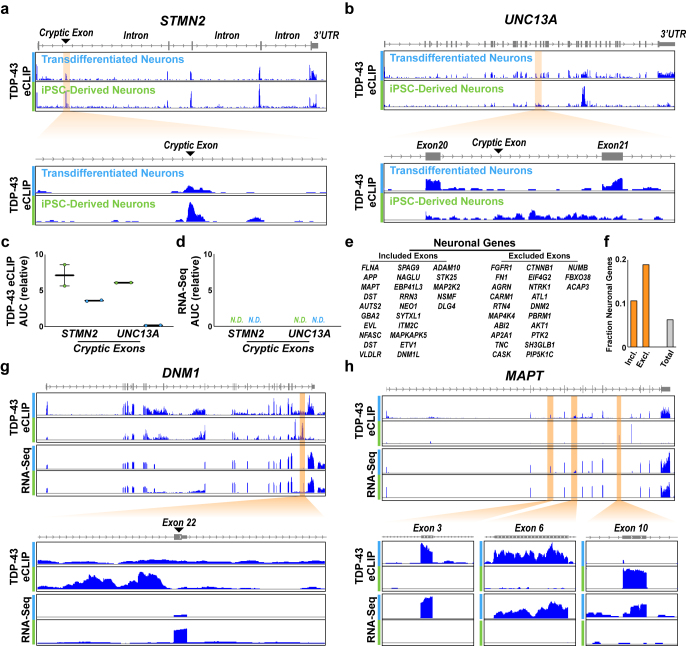
TDP-43 nuclear depletion causes alternative exon inclusion and exclusion. **a**, TDP-43 eCLIP binding intensities at the reported *STMN2* cryptic exon site in iPSC-diff.1 (green) and Tdiff.1 (blue) neurons from representative genomic tracks. **b**, Same as **a**, but for the *UNC13A* cryptic exon site. **c**, Area under the curve of TDP-43 binding intensity at the cryptic exons for *STMN2* and *UNC13A* in transdifferentiated (blue) and iPSC-derived (green) neurons (n = 2 replicates). The centerline depicts the median and the error bars denote the range of all values (n = 2 eCLIP replicates). **d**, Same as **c**, but for RNA-seq. ND, not detected. **e**, List of alternatively spliced genes at TDP-43 binding windows falling within the gene ontology set for neuronal development (GO:0048666). **f**, Fraction of alternatively spliced genes that are neuronal relative to the total alternatively spliced TDP-43 genes. The total number of neuronal genes (GO:0048666) is plotted relative to the total number of protein-coding genes for reference. **g**, TDP-43 eCLIP binding intensities and RNA-seq read density tracks at the *DNM1* gene for iPSC-derived (green) and transdifferentiated (blue) neurons. **h**, Same as **g**, but for the *MAPT* gene.

TDP-43 mediates the alternative splicing of hundreds of transcripts, and it was previously reported that loss of nuclear TDP-43 leads to the expression of *STMN2* and *UNC13A* cryptic exons in patients with ALS^27–29^. Therefore, we next tested whether splicing of these TDP-43 targets was also dysregulated in aged neurons. We performed enhanced cross-linking and immunoprecipitation (eCLIP)^30^ of TDP-43 in transdifferentiated and iPSC-derived neurons to determine TDP-43 binding sites on RNA. Consistent with the nuclear depletion of TDP-43 that we observed, TDP-43 interacted with substantially fewer introns in transdifferentiated neurons despite retaining its canonical GU-rich binding motif^31^ (Fig. 2d–e). This reduced intronic binding coincided with a >2.5-fold depletion in TDP-43 binding to the ALS-linked cryptic exon splice sites in *STMN2* and *UNC13A* (Extended Data Fig. 4a–c), although we did not detect cryptic exon expression (Extended Data Fig. 4d). We mapped TDP-43-bound introns to differentially spliced transcripts in aged neurons, and we observed significant changes in exon inclusion or exclusion events of approximately 500 transcripts, many of which were neuronal cytoskeleton proteins (Fig. 2f,g and Extended Data Fig. 4e,f). For example, reduced TDP-43 binding at the intronic site between exons 35 and 36 of the neuromuscular junction protein *AGRN* led to the exclusion of these exons in transdifferentiated neurons (Fig. 2h). We observed a similar exon exclusion event in *DNM1* (Extended Data Fig. 4g). Both *AGRN* and *DNM1* are previously reported targets of TDP-43 splicing^32^, and dysregulation of *AGRN* is implicated in ALS^33^. Conversely, increased TDP-43 binding at exons 3 and 6 in the dementia-associated gene *MAPT* led to retention of those exons in transdifferentiated neurons, which is the expected alternative splicing pattern of *MAPT* in adults^34,35^ (Extended Data Fig. 4h).

**Extended Data Fig. 5:**
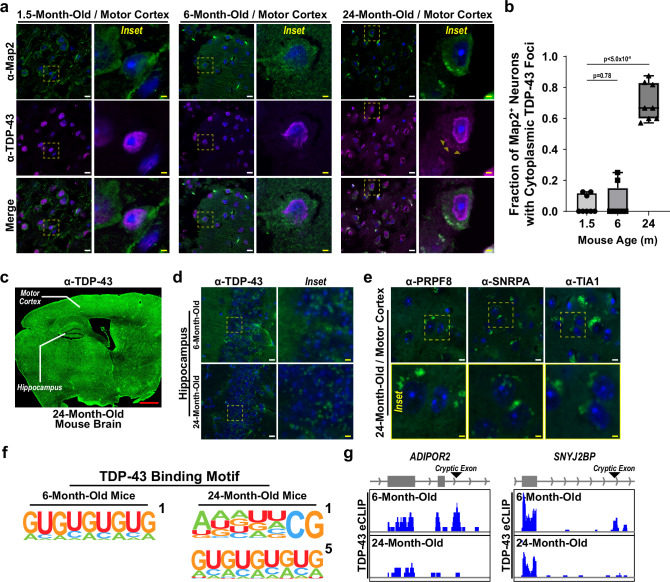
TDP-43 mislocalization is aging-dependent in mice. **a**, Confocal fluorescence images of TDP-43 (magenta), Map2 (green) and DAPI (blue) in 1.5-, 6- and 24-month-old mouse motor cortices. The inset region is denoted by the yellow dashed box. White scale bar = 10 μm; yellow scale bar = 2 μm. **b**, Quantification of the proportion of cells with cytoplasmic TDP-43 foci in Map2^+^ neurons from the images in **a** (n = 8 replicates from N = 2 mice per cohort). The box plot represents the 25th to 75th percentile of data values, and the centerline is the median. The error bars denote the range of all individual values. Statistics were calculated using a two-tailed Welch’s t test. **c**, Z-maximum projection of a tiled confocal fluorescence image of the 24-month-old brain stained for TDP-43 (green). Red scale bar = 1 mm. **d**, Z-maximum projection of confocal fluorescence image of TDP-43 (green) and DAPI (blue) in the hippocampus of 6- and 24-month-old mouse brains. White scale bar = 10 μm; yellow scale bar = 2 μm. **e**, Confocal fluorescence images of the indicated splicing proteins in the motor cortex of 24-month-old mouse brain slices. Scale bar = 10 μm. **f**, The top RNA-binding motifs for TDP-43 eCLIP in the 6- and 24-month-old mouse brain (n = 2 replicates). For 24-month-old mice, the top (‘1’) and 5th most significant (‘5’) motifs are shown. **g**, TDP-43 eCLIP binding intensities from representative genomic tracks at the indicated cryptic exon sites in *ADIPOR2* (left) and *SYNJ2BP*, both of which contain previously reported TDP-43 cryptic exons^32^.

To confirm that nuclear depletion of TDP-43 occurred as a function of aging, we tested whether the TDP-43 phenotypes we observed in transdifferentiated neurons were similar in aged wild-type mice (Fig. 2I). Immunofluorescence of 1.5-, 6- and 24-month-old mice confirmed that TDP-43 was mislocalized as a function of aging—neurons from the motor cortex of 1.5-month-old mice had strongly nuclear TDP-43, whereas the motor cortex neurons of 24-month-old mice TDP-43 contained cytoplasmic foci (Fig. 2j and Extended Data Fig. 5a–c). These aggregates were not present in either the hippocampus or motor cortex of the 6-month-old mice (Fig. 2j and Extended Data Fig. 5a–d). Like TDP-43, core spliceosome proteins like PRPF8 and SNRPA—and the alternative splicing protein TIA1—were mislocalized in aged mice (Extended Data Fig. 5e). TDP-43 eCLIP of the 1.5-, 6- and 24-month-old mouse cerebellum also revealed age-dependent loss of intronic binding (Fig. 2k) despite retaining the normal TDP-43 binding motif (Fig. 2l and Extended Data Fig. 5f). Consistent with our findings using transdifferentiated neurons, we observed reduced TDP-43 binding to reported cryptic exon sites in aged mice^32^ (Extended Data Fig. 5g). Together, our data demonstrates that mislocalization of TDP-43 and other splicing components is an aging-related process that alters the splicing of hundreds of transcripts, many of which are important for neuronal health.

### Aged neurons form chronic stress granules

Our TDP-43 AP-MS and immunofluorescence results indicated that spliceosome components assembled independently of stress granules, which are the typical repository for cytoplasmic RBPs during stress events (Fig. 2b and Extended Data Fig. 3g–l). Stress granules are biomolecular condensates that form in the cytoplasm due to activation of the integrated stress response^36^. One of the main functions of stress granules is the sequestration of mRNAs and RBPs until after the stress event is resolved^37,38^, and TDP-43 exclusion from stress granules is thought to lead to neurodegeneration-linked TDP-43 fibrillization^39^. Therefore, we tested whether dysregulation of the integrated stress response might be linked to TDP-43 mislocalization.

**Fig. 3:**
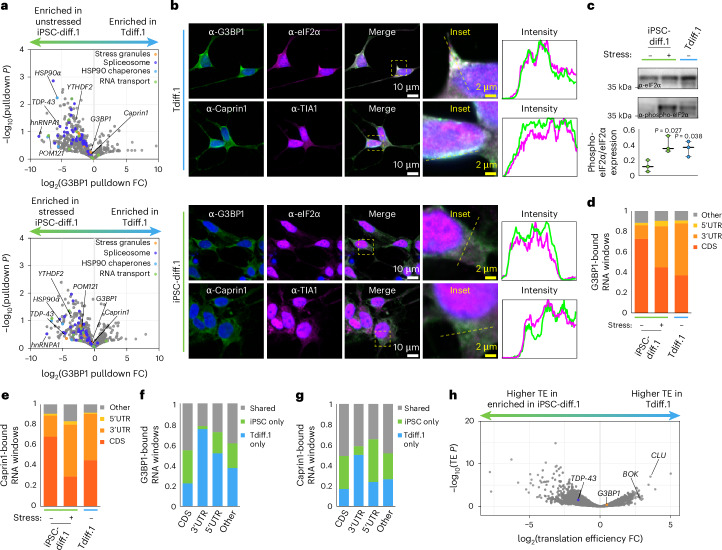
Aged neurons have chronic stress granules that repress translation. **a**, Volcano plots of G3BP1 pulldown and MS of all detected proteins in transdifferentiated neurons versus unstressed (top) or stressed (bottom) iPSC-derived neurons (*n* = 2 replicates). Detected proteins included in the following KEGG terms were highlighted: spliceosome (dark blue, hsa03040), stress granules (orange, hsa03019 + 10146), HSP90 chaperones (light blue, no KEGG pathway) and RNA transport (green, hsa03013). *P* values were calculated using a two-tailed Welch’s *t* test. **b**, Confocal fluorescence images of stress granule markers in transdifferentiated and iPSC-derived (iPSC-diff) neurons. The yellow boxes denote the inset region, and the yellow dashed lanes indicate the intensity profiles that were normalized and plotted in the last column. White scale bar = 10 μm; yellow scale bar = 2 μm. **c**, Top and middle, western blots of total and phosphorylated eIF2α for the indicated samples. Bottom, quantification of average phospho-eIF2α-to-total eIF2α expression for *n* = 3 blots, where the centerline denotes median and the error bands denote the range of data values; statistics were calculated using a two-tailed Welch’s *t* test comparing the indicated samples to the unstressed iPSC-derived neurons. **d**, Fraction of G3BP1-bound RNA windows detected by eCLIP (*n* = 2 replicates) within each of the indicated transcript elements for transdifferentiated and iPSC-derived neurons. **e**, Same as **d**, but for Caprin1-bound RNAs. **f**, Fraction of G3BP1-bound RNA windows detected in unstressed iPSC-derived neurons and/or transdifferentiated neurons. **g**, Same as **f**, but for Caprin1-bound RNAs. **h**, Volcano plot of translation efficiency of all detected transcripts using Ribo-seq in transdifferentiated and iPSC-derived neurons. CDS, coding sequence; TE, translation efficiency. Source data

**Extended Data Fig. 6:**
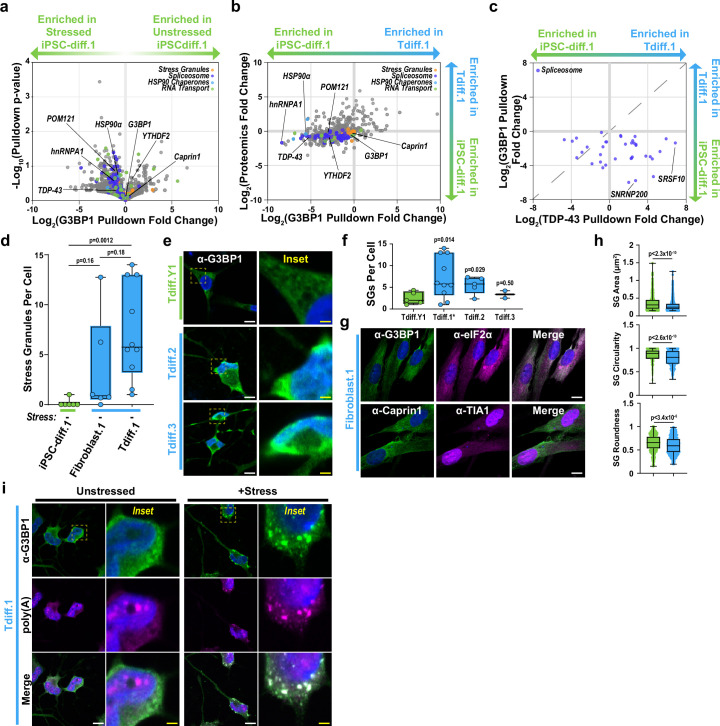
G3BP1 assembles into stress granules in aged neurons. **a**, Volcano plot of G3BP1 pulldown and mass spectrometry of all detected proteins in unstressed versus stressed iPSC-diff.1 neurons (n = 2 replicates). P values were calculated using a two-tailed Welch’s t test. **b**, Scatter plot of G3BP1 pulldown fold change plotted with the proteomic fold change for each detected protein (n = 2 replicates). **c**, Scatter plot of TDP-43 pulldown fold change versus G3BP1 pulldown fold change for all proteins falling within the spliceosome KEGG term (hsa03040). **d**, Quantification of the number of stress granules per cell in the indicated isogenic cell types (n = 6 replicates). The box plots represent the 25th to 75th percentile, the centerline denotes the median and the error bars denote the range of all data values. Statistics were calculated using a two-tailed Welch’s t test. **e**, G3BP1 and DAPI staining of various transdifferentiated neuron lines visualized by fluorescence confocal microscopy. The yellow box denotes the inset. White scale bar = 10 μm; yellow scale bar = 2 μm. **f**, Same as **d**, but for the additional Tdiff lines from **e**. Tdiff.1 was replotted from **d**. Statistics were calculated pairwise with the Tdiff.Y1 line using a two-tailed Welch’s t test. **g**, Fluorescence confocal images of primary fibroblasts stained for stress granule markers. White scale bar = 10 μm. **h**, Quantification of the average stress granule area, circularity and roundness in stressed Tdiff.1 (Fig. 2c/ED6I) versus iPSC-diff.1 neurons (Extended Data Fig. 3f) plotted as in ED6D (n > 100 stress granules in N = 5 replicates). Statistics were calculated using a two-tailed Welch’s t test. **i**, Confocal fluorescence images of unstressed and arsenite-stressed transdifferentiated neurons stained for G3BP1 (green) and poly(A) (magenta) via a dT FISH probe. Yellow boxes denote the inset region. White scale bar = 10 μm; yellow scale bar = 2 μm.

We performed AP-MS of G3BP1, which is a canonical stress granule marker. Consistent with our previous results from Fig. 2b, we found that spliceosome components were de-enriched from the G3BP1 interactome in aged neurons, especially TDP-43 (Figs. 2b and 3a and Supplementary Table 3). Acute stress treatment of iPSC-derived neurons caused a similar depletion of splicing proteins, indicating that transdifferentiated neurons might be basally stressed (Extended Data Fig. 6a). Other core stress granule proteins like Caprin1 were unchanged in transdifferentiated neurons, but auxiliary stress granule members like staufen-1 and staufen-2 were depleted, possibly due to their lower expression levels (Fig. 3a, Supplementary Table 3 and Extended Data Fig. 6b). The pulldown fold changes of spliceosome components for G3BP1 and TDP-43 were largely anticorrelated (Extended Data Fig. 6c), further indicating that they are distinct complexes in transdifferentiated neurons.

**Extended Data Fig. 7:**
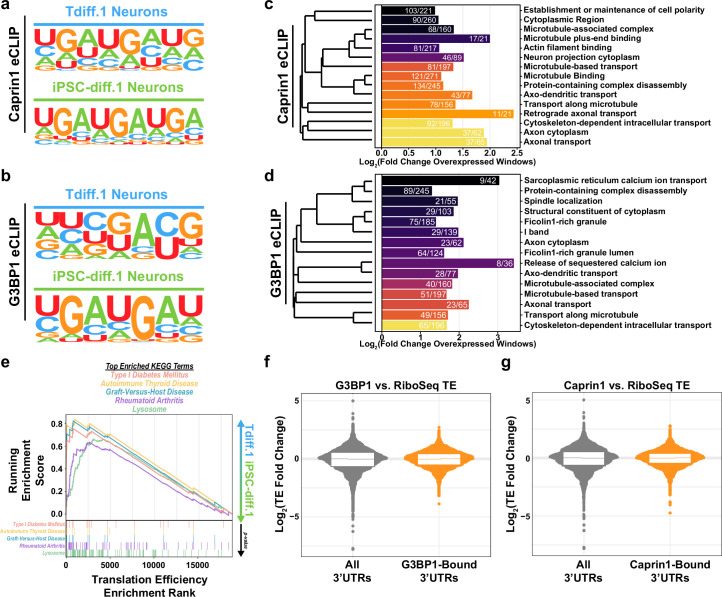
G3BP1 and Caprin1 binding do not impact translation efficiency. **a**, eCLIP binding motif of Caprin1 (n = 2 replicates). **b**, Same as **a**, but for G3BP1. **c**, Most significant gene ontology terms for Caprin1-bound transcripts detected by eCLIP. **d**, Same as **c**, but for G3BP1. **e**, Plot of the running enrichment score of the top KEGG terms for upregulated translation efficiency in Tdiff.1 neurons. **f**, Distribution of the log_2_(translation efficiency fold change) of all 3′ UTRs (gray) and G3BP1-bound 3′ UTRs (orange) in Tdiff.1 vs. iPSC-diff.1 neurons. The line within the box plot represents the median for the indicated population. **g**, Same as **f**, but for Caprin1-bound 3′ UTRs.

Under basal conditions, stress granule components are diffuse in the cytoplasm and will only assemble into condensates during stress events^40^. However, immunofluorescence revealed that core stress granule proteins like G3BP1 and Caprin1 pre-assembled into oblong, misshapen condensates in unstressed transdifferentiated neurons but not in iPSC-derived neurons (Fig. 3b and Extended Data Fig. 6d). G3BP1 condensates were observed in many transdifferentiated neuronal lines (Extended Data Fig. 6e,f), and they were specific to aged neurons because fibroblasts and young transdifferentiated neurons did not contain these inclusions (Extended Data Fig. 6d–g). Phosphorylated eIF2α is the canonical marker for activation of the integrated stress response^36^, and we indeed observed higher levels of phosphorylated eIF2α in transdifferentiated neurons relative to unstressed iPSC-derived neurons (Fig. 3c). The RNA-binding profile of G3BP1 and Caprin1 measured by eCLIP can also elucidate the stress status of cells; acute stress treatment causes stress granule proteins to bind more 3′ untranslated regions (UTRs) rather than coding (CDS) RNA sequences, and we found elevated 3′ UTR binding in transdifferentiated neurons without stress treatment (Fig. 3d,e). Interestingly, the 3′ UTR sequences that were bound by G3BP1 and Caprin1 in transdifferentiated neurons were mostly unique and were not bound in unstressed iPSC-derived neurons (Fig. 3f,g). The RNA-binding motif of Caprin1 was unchanged in aged neurons (Extended Data Fig. 7a,b), suggesting that these cryptic 3′ UTR sequences were likely exposed due to chronic stress rather than a shift in RNA-binding preference by the stress granule protein. G3BP1- and Caprin1-bound RNAs were enriched for neuronal transcripts (Extended Data Fig. 7c,d). Our data indicate that aged neurons are chronically stressed because of their unique, stress-associated phenotypes even at baseline conditions. Therefore, we refer to the stress granule-like inclusions in these aged neurons as chronic stress granules.

The formation of stress granules is associated with stalled translation^36^, and 3′ UTR binding by RBPs can also regulate translation of specific mRNAs^41^. Therefore, we used ribosome profiling sequencing (Ribo-seq)^42^ to determine the translation efficiency of stress granule-associated transcripts. In transdifferentiated neurons, thousands of transcripts had lower translation efficiency (Fig. 3h and Extended Data Fig. 7e). However, G3BP1- and Caprin1-bound transcripts had no shift in translation efficiency (Extended Data Fig. 7f,g), suggesting that chronic stress granules were not directly regulating translation in aged neurons. Moreover, we found that poly(A) RNA only accumulated into chronic stress granules in transdifferentiated neurons upon exogenous stress treatment (Extended Data Fig. 6i), and these granules were usually smaller and misshapen, indicating a reduction in fusion events^43^, relative to the canonical stress granules formed in iPSC-derived neurons (Extended Data Fig. 6h). Together, our data indicate that chronic, gel-like stress granules form in aged neurons, but they do not functionally alter translation.

### HSP90α activity antagonizes stress granule resolution

**Fig. 4:**
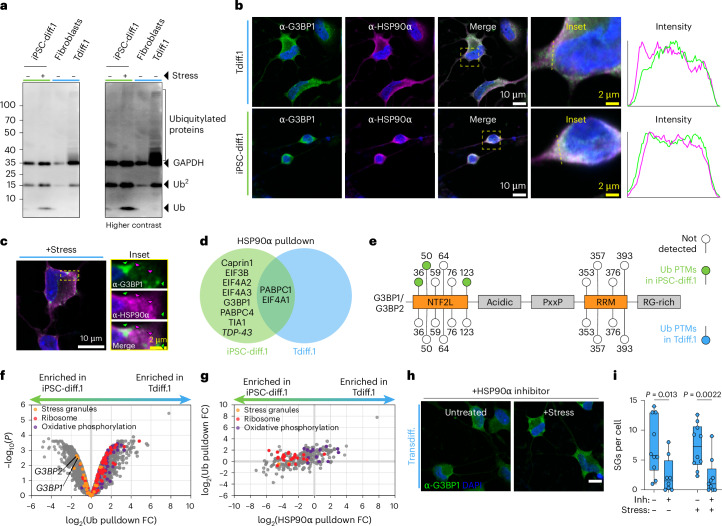
HSP90 activity antagonizes stress granule resolution in aged neurons. **a**, Representative western blot of ubiquitylated proteins in the indicated neurons. **b**, Confocal fluorescence images of G3BP1 and HSP90α in unstressed transdifferentiated and iPSC-derived neurons. The yellow boxed region denotes the inset, and the yellow dashed line denotes the region where the intensity of G3BP1 and HSP90α was plotted. White scale bar = 10 μm; yellow scale bar = 2 μm. **c**, Airyscan fluorescence images of transdifferentiated neurons stressed with sodium arsenite for 1 h and stained for G3BP1 and HSP90α. White scale bar = 10 μm; yellow scale bar = 2 μm. **d**, Venn diagram of stress granule proteins detected by HSP90α pulldown and MS (*n* = 2 replicates) in iPSC-derived and/or transdifferentiated neurons. **e**, Schematic representation of Ub modifications detected by TUBE pulldown and MS (*n* = 3 replicates) in iPSC-derived neurons (upper lollipops in green) and transdifferentiated neurons (lower lollipops in blue). Unshaded circles denote that the Ub modification was not detected. **f**, Volcano plot of the TUBE pulldown and MS results in iPSC-diff.1 and Tdiff.1 lines (*n* = 3 replicates). *P* values were calculated using a two-tailed Welch’s *t* test. Orange dots represent stress granule proteins, red dots denote ribosomal proteins and purple dots denote oxidative phosphorylation proteins. **g**, Same as **f**, but for HSP90α pulldown versus TUBE pulldown efficiency. **h**, Confocal fluorescence images of G3BP1 (green) and DAPI (blue) in unstressed or stressed transdifferentiated neurons treated with an HSP90α inhibitor for 24 h. **i**, Quantification of the average number of stress granules per cell in **h** (*n* = 10 replicates). The box plot denotes the range from the 25th to the 75th percentile of data values, where the middle line is the median; the error bars denote the range of all data values. Statistics were calculated using a two-tailed Welch’s *t* test. Ub, ubiquitin; SGs, stress granules.

**Extended Data Fig. 8:**
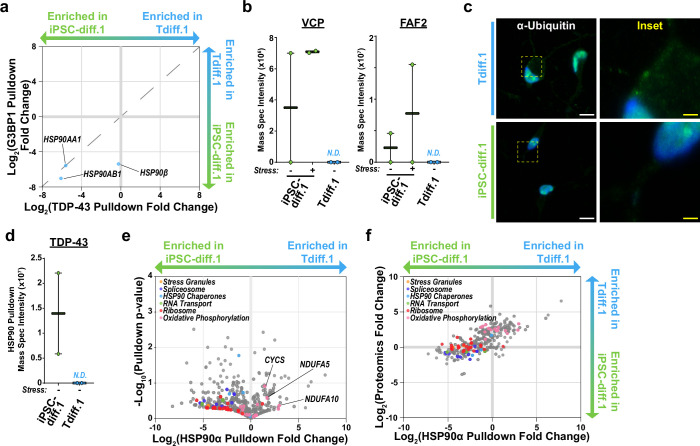
Cytoplasmic ubiquitylation is increased in transdifferentiated neurons. **a**, Scatter plot of TDP-43 pulldown fold change versus G3BP1 pulldown fold change for all detected HSP90 chaperones (n = 2 replicates). **b**, Raw mass spectrometry intensity of two proteins required for stress granule dissolution (VCP and FAF2) detected by G3BP1 pulldown (n = 2 replicates). The centerline denotes the median. ND, not detected. **c**, Confocal fluorescence images of ubiquitin in transdifferentiated iPSC-derived neurons. The yellow box denotes the inset. White scale bar = 10 μm; yellow scale bar = 2 μm. **d**, Raw mass spectrometry intensity of TDP-43 detected by HSP90α pulldown (n = 2 replicates). The centerline denotes the median. ND, not detected. **e**, Volcano plot of HSP90α pulldown and mass spectrometry of all detected proteins in transdifferentiated versus unstressed iPSC-derived neurons (n = 2 replicates). Detected proteins included in the following KEGG terms were highlighted—spliceosome (dark blue, hsa03040); stress granules (orange, hsa03019 + 10146); HSP90 chaperones (light blue, no KEGG pathway); RNA transport (green, hsa03013); ribosome (red, hsa03008); oxidative phosphorylation (pink, hsa00190). **f**, Scatter plot of HSP90α pulldown fold change plotted with the proteomic fold change for each detected protein (n = 2 replicates). Proteins were highlighted as described in **e**.

The presence of chronic stress granules in aged neurons indicated that the dissolution of stress granules may be dysregulated, which in turn may exacerbate the dysregulated RNA metabolism we observed in Figs. 1 and 2. Stress granule resolution is mediated by ubiquitination of G3BP1, which is then recognized by VCP, a multifunctional protein that is involved in many ubiquitin-related processes^44^. ATPase activity of HSP90α, which also regulates the ubiquitinome, is required for proper resolution of stress granules^45^; HSP90 and VCP work together to clear misfolded proteins, leading to undetectable poly-ubiquitin chains in normal cells (Fig. 4a)^46^. We noticed in our affinity pulldowns of G3BP1 and TDP-43 that HSP90α and its subunits were among the most depleted interacting proteins in aged neurons, potentially indicating that dysregulation of this heat shock protein was causing poor stress granule resolution (Figs. 2b and 3a, Supplementary Table 4 and Extended Data Fig. 8a). HSP90α foci were also visibly distinct from chronic and arsenite-mediated stress granules in aged neurons (Fig. 4b,c). Moreover, VCP and its cofactor FAF2 were entirely absent from the G3BP1 interactome in transdifferentiated neurons (Extended Data Fig. 8b). Therefore, we hypothesized that altered stress granule component ubiquitylation contributed to the lack of stress granule dissolution.

We first measured the status of the ubiquitinome. As determined by western blotting and immunofluorescence, ubiquitination was broadly increased in transdifferentiated neurons (Fig. 4a and Extended Data Fig. 8c), which may be driving the integrated stress response through the accumulation of unfolded proteins^47^. Given that HSP90α chaperones hundreds of protein targets and is depleted from the stress granule interactome, we reasoned that its activity may be responsible for chronic stress granule presence. We then examined the HSP90α interactome, which confirmed the reduced interaction between HSP90α and stress granule proteins in aged neurons (Figs. 2b, 3a and 4d). HSP90α also did not bind to TDP-43 in transdifferentiated neurons despite interacting in iPSC-derived neurons (Extended Data Fig. 8d). The HSP90α interactome of aged neurons was enriched with several proteins required for oxidative phosphorylation, including cytochrome c and the ubiquinone complex (Extended Data Fig. 8e). Notably, these oxidative phosphorylation proteins were highly expressed in aged neuron data despite having normal transcript expression (Fig. 1k,l, Extended Data Fig. 8f and Supplementary Table 5), suggesting a slow turnover rate that is perhaps caused by poor HSP90α activity.

To test if HSP90α might be stalled on ubiquitylated mitochondrial proteins, we performed a ubiquitin pulldown and MS in both transdifferentiated and iPSC-derived neurons. Our results supported our hypothesis in the following three ways: (1) our ubiquitin pulldown in transdifferentiated neurons failed to directly detect any of the G3BP1/G3BP2 ubiquitin modifications that are required for stress granule turnover^44^ (Fig. 4e), (2) the pulldown found that G3BP1/G3BP2 were depleted from the ubiquitin interactome in transdifferentiated neurons (Fig. 4f) and (3) the mitochondrial proteins that bind HSP90α strongly in transdifferentiated neurons were also highly enriched in our ubiquitin pulldown (Fig. 4f,g). Together, these results indicate that stress granule turnover is deprioritized by protein chaperones and the ubiquitin machinery in favor of mitochondrial proteins.

Because HSP90α has ATPase-dependent foldase and ATPase-independent holdase activity^48–50^, we treated aged neurons with the HSP90α inhibitor ganetespib to bias the cells toward the holdase activity. We surprisingly found that HSP90α inhibitor treatment was sufficient to significantly reduce both chronic and arsenite-mediated stress granule formation (Fig. 4h,i), further indicating that HSP90α dysregulation contributes to the chronic stress observed in aged neurons.

### Chronic stress granules dampen the response to acute stress

**Fig. 5:**
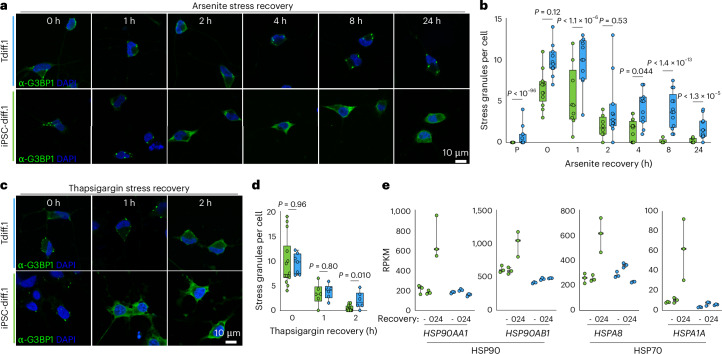
Transdifferentiated neurons fail to resolve stress granules after acute stress treatment. **a**, Confocal fluorescence images of G3BP1 (green) and DAPI (blue) in transdifferentiated and iPSC-derived neurons at the indicated time points after 1 h of sodium arsenite treatment. Scale bar = 10 μm. **b**, Quantification of the average number of stress granules (*n* = 3 replicates) for transdifferentiated (blue) and iPSC-derived (green) neurons in **a**. The box plot denotes the range from the 25th to the 75th percentile of data values, where the middle line is the median; the error bars denote the range of all data values; statistics were calculated using Welch’s *t* test. **c**, Same as **a**, but for thapsigargin stress recovery. **d**, Same as **b**, but for thapsigargin stress recovery. **e**, Average RNA-seq RPKM for the indicated heat shock proteins in iPSC-derived (green) and transdifferentiated (blue) neurons (*n* = 3 replicates) at the indicated time points before (−), immediately after (0) or 24 h after stress (24). The centerline denotes the median, and the error bars denote the range of values. NS, not significant; P, prestress; RPKM, reads per kilobase million.

**Extended Data Fig. 9:**
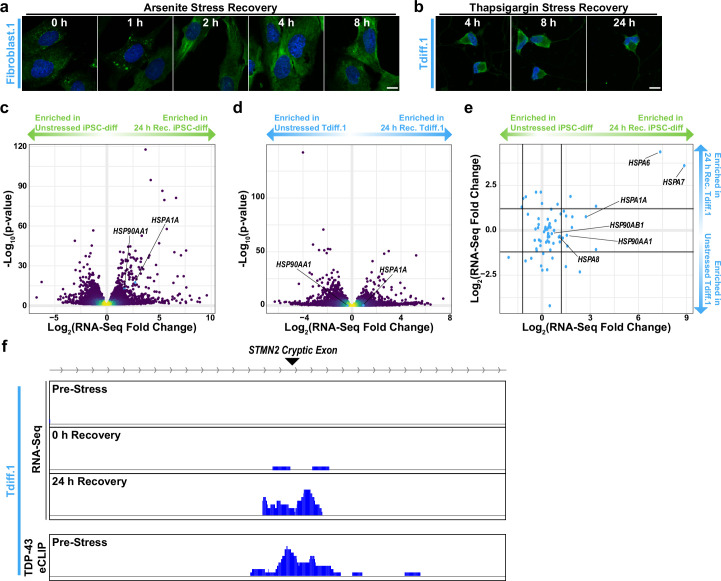
Aged neurons fail to activate heat shock proteins in response to arsenite stress. **a**, Confocal fluorescence images of G3BP1 (green) and DAPI (blue) in fibroblasts (fibroblast.1) treated with arsenite for 1 h and allowed to recover for the indicated amount of time. Scale bar, 10 μm. **b**, Confocal fluorescence images of G3BP1 (green) and DAPI (blue) in Tdiff.1 neurons treated with thapsigargin for 1 h and allowed to recover for the indicated time points. Scale bar, 10 μm. **c**, Volcano plot of RNA-seq differential expression in unstressed iPSC-derived neurons compared to iPSC-derived neurons treated with arsenite for 1 h and recovered for 24 h (n = 3 replicates). P values were calculated using a Wald test. The indicated heat shock proteins are highlighted in light blue; points with high clustering are green/yellow. **d**, Same as **c**, but for transdifferentiated neurons (n = 3 replicates). **e**, Scatter plot of RNA-seq fold change of all heat shock protein transcripts for unstressed vs. recovered iPSC-derived and transdifferentiated neurons (n = 3 replicates). **f**, Representative genomic tracks of RNA-seq (top three tracks) and eCLIP (bottom track, reproduced from Extended Data Fig. 4a) at the *STMN2* cryptic exon locus before stress (pre-stress), immediately after stress (0 h recovery) and after stress (24 h recovery) in Tdiff.1 neurons.

The chronic activation of the integrated stress response in aged neurons may inhibit their effective response to new stress stimuli. For example, extended treatment with low levels of exogenous stressors was shown to dysregulate the integrated stress response in neuroblastoma cells^51^ and cause TDP-43 aggregation in motor neurons^52^. Therefore, we treated iPSC-derived and transdifferentiated neurons with sodium arsenite for 1 h and allowed them to recover for up to 24 h. iPSC-derived neurons resolved ~50% of arsenite-induced stress granules within 2 h (Fig. 5a,b). Primary fibroblasts had similar stress granule recovery kinetics (Extended Data Fig. 9a). By contrast, transdifferentiated neurons responded poorly to stress and had elevated stress granule retention even 24 h postarsenite treatment (Fig. 5a,b). Poor stress granule resolution in transdifferentiated neurons was also observed in response to treatment with an endoplasmic reticulum stressor, thapsigargin (Fig. 5c,d and Extended Data Fig. 9b). Given that arsenite and thapsigargin typically activate the stress response via different eIF2α kinases, our data indicate that chronic phosphorylation of eIF2α (Fig. 4c) renders aged neurons incapable of efficiently recovering from distinct types of acute stress.

To determine whether acute stress also affected downstream functions of the integrated stress response, we performed RNA-seq of iPSC-derived and transdifferentiated neurons immediately after arsenite treatment and after 24 h recovery from arsenite. It was previously reported that various heat shock proteins were activated following stress treatment and recovery^53^, and we observed a similar increase in the transcript levels of HSP70 and HSP90 components following stress treatment in iPSC-derived neurons (Fig. 5e and Extended Data Fig. 9c), including *HSPA1A*, which is only transcribed in response to stress^54^. However, transdifferentiated neurons failed to activate transcription of HSP70 and HSP90 chaperones (Fig. 5e and Extended Data Fig. 9d,e). Only *HSPA6* and *HSPA7* were strongly activated in transdifferentiated neurons, but upregulation of these genes was even more pronounced in iPSC-derived neurons (Extended Data Fig. 9e). In addition, we found that expression of the *STMN2* cryptic exon was activated in transdifferentiated neurons 24 h following arsenite treatment (Extended Data Fig. 9f). Together, our results demonstrate that aged neurons fail to properly respond to and recover from acute stress treatments, indicating a lack of resiliency that may underlie aging-related neuronal death.

### The aging human brain has dysregulated RNA biology

**Fig. 6:**
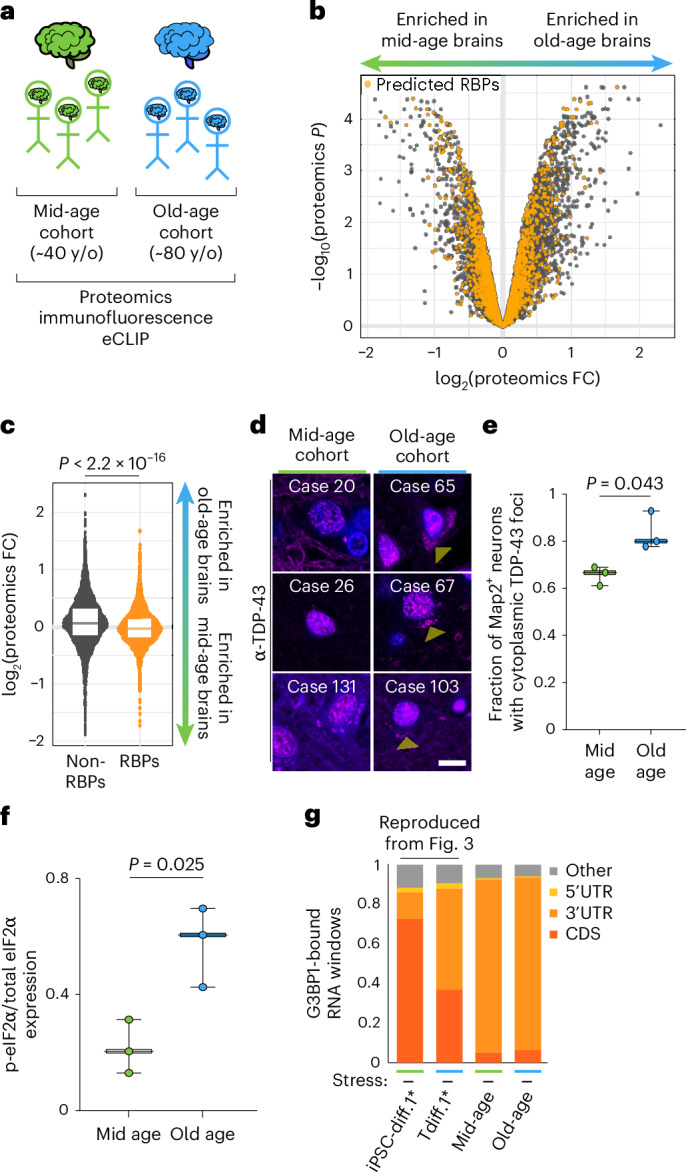
RNA biology is dysregulated in the aging human brain. **a**, Schematic representation of the aged human brain cohorts. **b**, Volcano plot of all proteins detected by MS (*n* = 2 replicates per tissue sample; *n* = 3 samples per cohort) in the mid-age and old-age human brains. Predicted RBPs are highlighted in orange^62^. **c**, Violin plot of all non-RBPs (gray) and RBPs (orange) expression in the mid-age and old-age brain cohorts. The box plot denotes the range of the 25th and 75th percentiles of all data points, and the centerline denotes the median. The *P* value was calculated using a two-tailed Welch’s *t* test. **d**, IF of TDP-43 (magenta) and DAPI (blue) in the aged human brain cohorts. Yellow arrowheads denote cytoplasmic TDP-43 foci. **e**, Quantification of the proportion of Map2^+^ neurons with cytoplasmic TDP-43 foci in each of the human brain cohorts (*n* = 8 replicates per tissue sample; *n* = 3 samples per cohort). The centerline denotes the median, and the error bands denote the range of data values. Statistics were calculated using a two-tailed Welch’s *t* test. **f**, Quantification of phospho-eIF2a expression relative to total eIF2α signal (Extended Data Fig. 10b, blot). The centerline denotes the median, and the error bands denote the range of data values. Statistics were calculated using a two-tailed Welch’s *t* test. **g**, Fraction of G3BP1-bound RNA windows detected by eCLIP (*n* = 2 replicates for cell samples, which are replotted from Fig. 3; *n* = 3 samples per brain cohort, which are treated as replicates for this experiment) within each of the indicated transcript elements. y/o, year old.

Although transdifferentiated neurons are a powerful model to study aging, we sought to verify whether key phenotypes that we observed in cell culture are also present in aged human brains. To this end, we repeated several experiments—MS, immunofluorescence and eCLIP—in cohorts of aged brain tissue; we used the frontal cortex, which should mirror the cortical neurons we generated via transdifferentiation. Our cohorts consisted of three ‘mid-age’ brains (30–50 years old) and three ‘old-age’ brains (80–90 years old; Supplementary Table 6 and Fig. 6a). MS of lysed brain tissue revealed that there were several thousand proteins that were substantially upregulated and downregulated due to aging (Fig. 6b); the upregulated pathways primarily encompassed mitochondrial gene sets like oxidative phosphorylation and aerobic respiration (Supplementary Table 7). By contrast, RNA pathways were largely depleted due to aging (Supplementary Table 7), and RBPs were expressed at significantly lower levels than all other proteins in our old-age cohort relative to the mid-age cohort (Fig. 6c). This directly recapitulates the phenotype that we observed in transdifferentiated neurons compared to their isogenic iPSC-derived control neurons (Fig. 1i,j).

**Extended Data Fig. 10:**
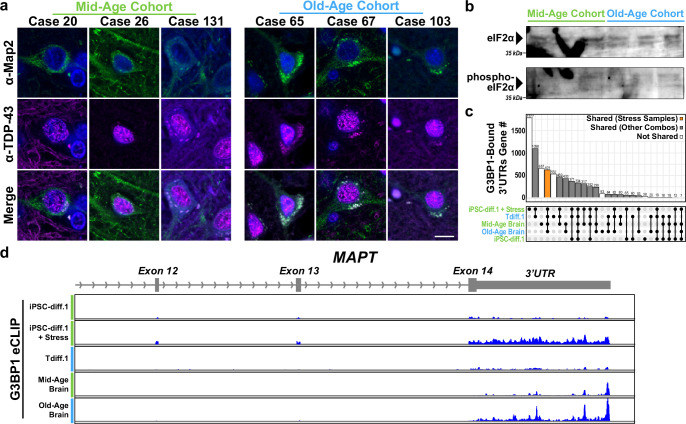
The aging human brain has cytoplasmic TDP-43 foci. **a**, Confocal immunofluorescence images of frontal cortex tissue from aged cohorts of human brains. Green is Map2, magenta is TDP-43 and blue is DAPI. White scale bar = 10 μm. The TDP-43 images are reproduced from Fig. 6d. **b**, Western blot of eIF2α and phospho-eIF2α in aged human brain samples (N = 3 samples per cohort). Quantification is provided in Fig. 6f. **c**, Upset plot of shared 3′ UTR binding sites (enrichment score >3) among the indicated G3BP1 eCLIP samples. UTRs were grouped by genes. The orange bar indicates the 3′ UTRs that were detected in all stressed samples, gray bars are other combinations of 3′UTRs detected in multiple genes and white bars denote 3′UTRs that were detected in only one sample. **d**, Representative genomic tracks of G3BP1 binding to the *MAPT* 3′ UTR in cultured neurons (top three tracks, original data in Fig. 3) and brain tissue (bottom two tracks, original data in Fig. 6). The exons and 3′UTR are indicated on the gene. Source data

Given the similar RBP depletion in both cultured neurons and tissue, we tested whether TDP-43 was also basally mislocalized in the aging human brain. Immunofluorescence staining of TDP-43 in human brain tissue indeed showed an age-dependent increase in cytoplasmic TDP-43 foci (Fig. 6d–e and Extended Data Fig. 10a), which is a finding that we observed in transdifferentiated neurons and in the aging mouse brain (Fig. 2).

Finally, we tested whether the aged human brain also displayed key phenotypes of molecular stress—elevated levels of phospho-eIF2α and increased 3′ UTR binding by G3BP1. As noted above, eIF2α phosphorylation initiates the formation of stress granules, and we found that the old-age brain cohort expressed significantly higher levels of phospho-eIF2α relative to the mid-age brain cohort (Fig. 6f and Extended Data Fig. 10b). In addition, G3BP1 eCLIP of the mid- and old-age brain cohorts displayed the predominantly 3′ UTR binding found in stressed conditions (Fig. 6g). Many 3′ UTR binding sites were shared between the brain samples and our cultured neurons (Extended Data Fig. 10c), which included known G3BP1 binding targets in neurons like *MAPT*^55^ (Extended Data Fig. 10d). Together, our data demonstrates that our findings in transdifferentiated neurons are largely recapitulated in the aging human frontal cortex.

## Discussion

Aging is one of the primary risk factors for neurodegeneration, and our study demonstrates that aging alone mechanistically and functionally destabilizes RNA metabolism in neurons. Our conclusions are drawn from several lines of orthogonal evidence in transdifferentiated neurons compared to isogenic iPSC-derived neurons (Figs. 1–5) with further validation in a mouse model (Fig. 2i–l) and in the human brain (Fig. 6). We demonstrate that transdifferentiation successfully generates aged neurons that express key neuronal markers and can fire both spontaneous and induced action potentials (Extended Data Fig. 1). Despite being a two-dimensional cell culture model that does retain expression of some fibroblast markers like COL1A1 (Extended Data Fig. 2a), the results from transdifferentiated neurons are largely recapitulated in the aged mouse cortex and aged human frontal cortex.

However, we do note a few differences, which are as follows: (1) human brain tissue has a lower, albeit still significant, degree of RBP depletion due to aging; (2) both human and mouse brains retain substantial levels of nuclear TDP-43 in their neurons and (3) the G3BP1 eCLIP binding in the human brains does not change as a function of aging. There are several explanations for these incongruencies. First, our cell culture model is a comparison between fetal neurons and aged neurons; fetal neurons are very different from even the youngest mouse and human brains we assay. Second, we are encumbered by the limitations of working with tissue, including the time between death and dissection, which may lead to degradation of unstable RNAs and cellular structures^56^. Third, the auxiliary glia cells of the brain may help clear protein aggregates in unhealthy neurons of a human or mouse, but this does not occur in our monoculture neurons^57^. Still, all our results clearly demonstrate that the RNA biology of an aged neuron is indeed ailing.

The proteomes of transdifferentiated neurons and human brains demonstrate that thousands of canonical RBPs are depleted in aged neurons, especially those involved in splicing and RNA transport. Intriguingly, the mRNA transcripts encoding RBPs are not depleted, indicating that the RBPs are perhaps being degraded more rapidly in aged neurons. This increased turnover could be due to a failure to localize to the proper region of the neuron. Indeed, dysregulation of nucleocytoplasmic transport was previously reported in transdifferentiated neurons^58^, and we likewise find that the depleted splicing proteins, including the ALS- and dementia-associated RBP TDP-43, are mislocalized to the cytoplasm of aged neurons.

Mislocalization of TDP-43 is thought to be an initiating step in the pathogenesis of ALS, yet the cause of TDP-43 nuclear depletion to this point was unclear^11^; our results indicate that aging is sufficient to cause TDP-43 mislocalization, which may act as the first insult driving neurons toward neurodegeneration. Consistent with this hypothesis, TDP-43 mislocalization in aged neurons decreases binding to neurodegeneration-linked cryptic exon sites. The next step of TDP-43-mediated pathogenesis is thought to be aggregation into fibrils, which is usually antagonized by stress granules^39^. Our recent report suggests that splicing proteins like SNRNP200 are recruited to stress granules in human embryonic kidney (HEK) cells^59^; however, in aged neurons, splicing proteins including TDP-43 form complexes that are distinct from stress granules.

**Fig. 7:**
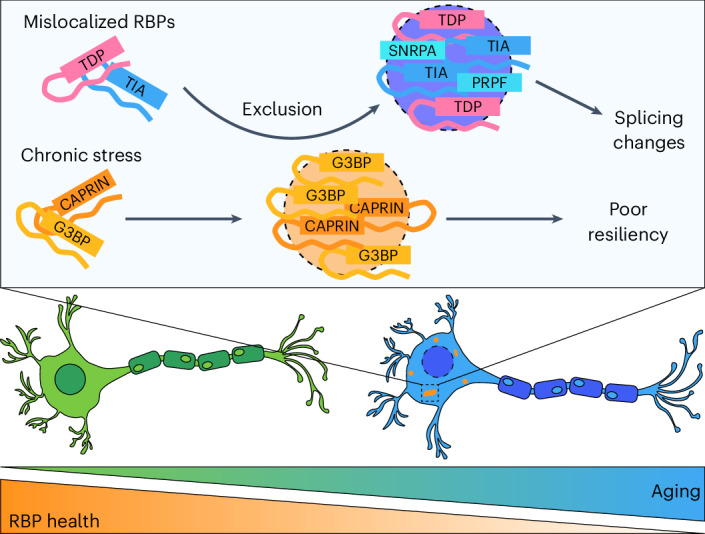
Schematic representation of dysregulated RNA biology in aging neurons. Chronic activation of the stress response in aged neurons leads to physical demixing between stress granules and other RBPs, which functionally impacts splicing in aged neurons and decreases resiliency to acute stress.

The chronic stress granules that form in aged neurons are compositionally distinct from canonical stress granules. Key RBPs are depleted from chronic stress granules, including RNA transport and splicing proteins, as mentioned above. Moreover, the physical properties of chronic stress granules appear to be gel-like, given their small area and oblong shape, which may explain why there is physical demixing between phase-separated stress granules and TDP-43 (refs. ^60,61^). The gel-like properties of chronic stress granules may arise from a prolonged inability to dissolve and may hinder recruitment of other RBPs even during new stress events. Together, we propose a model in which chronic, aging-related stress leads to TDP-43 exclusion from stress granules, which functionally destabilizes TDP-43-mediated splicing in aged neurons (Fig. 7).

The failure to respond to acute stress may further exacerbate the aging-mediated dysregulation of RNA biology we described. Given that we detected depletion of HSP90α from stress granules, the lack of HSP90α activation in response to acute stress treatment likely increases the relative burden of unfolded protein stress on aged neurons. In fact, the depletion of HSP90α from stress granules in aged neurons may result from one—or many—stress events that occurred before culturing of the primary fibroblasts. It is possible that resiliency to such stress events could be increased by exogenously rescuing chaperone activity in aging neurons. Therefore, reversing chronic stress may be a viable therapeutic avenue to prevent neurodegeneration and should be a focus of future efforts to promote the healthy aging of neurons.

## Methods

### Lentiviral production

Lenti-X 293T (Takara Bio, 632180) cells were cultured in DMEM (high glucose; Life, 11965118) with 10% (vol/vol) FBS (Life, 26140079) and passaged with 1:10 split ratios at 90% confluency using TrypLE Express (Life, 12604013). Within 24 h of a fresh passage, Lenti-X 293Ts were cotransfected with psPAX2 (Addgene, 12260), pMD2.G (Addgene, 12259) and pLVX-UbC-rtTA-Ngn2:2A:Ascl1 (UNA; Addgene, 127289) or pLVX-UbC-rtTA-Ngn2:2A:EGFP (rtTA-Ngn2; Addgene, 127288) at a 1:1:2 mass ratio using the JetPEI transfection system (VWR, 89129-916). The supernatant fraction of transfected Lenti-X 293T cells was collected 48 h, 72 h and 96 h post-transfection and pooled at 4 °C. Lenti-X concentrator (Takara Bio, 631232) was added to the collected virus, incubated at 4 °C for 30 min and centrifuged at 15,00*g* for 45 min at 4 °C. The lentivirus-containing pellet was resuspended using 1× dPBS (Life, 13190250) in 1/100 of the original volume of the pooled supernatant fraction. Lentiviral titer was measured using Lenti Go-Stix (Takara Bio, 631280) and aliquoted at the desired titer for long-term storage at −80 °C.

### Fibroblast cell culture and transduction

Primary fibroblasts originating from antemortem patients (Supplementary Table 8) were cultured on uncoated plastic six-well plates (Thermo Fisher Scientific, FB012927) in TFM (DMEM, high glucose (Life, 11965118), 15% (vol/vol) FBS (Life, 26140079) and 1× MEM NEAA (Thermo Fisher Scientific, 11140050)) at 37 °C and 5% CO_2_ with media changes every 2–3 days. Fibroblasts were passaged at 90–100% confluency with a 1:2 or 1:3 split ratio using TrypLE Express (Life, 12604013). Long-term fibroblast stocks were stored in 90% FBS with 10% (vol/vol) DMSO (Sigma, D2650-100ML) in liquid nitrogen. To generate transdifferentiation-competent fibroblasts, 80% confluent cells were transduced in 500 μl TFM with lentivirus containing the UNA expression vector in 5 μg ml^−1^ polybrene (Sigma, TR-1003-G). Following incubation at 37 °C with 5% CO_2_ for 8 h, the transduction volume was increased to 2 ml, and the fibroblasts were incubated with virus for an additional 40 h. To select for fibroblasts containing the UNA vector, the fibroblasts were treated with 1 μg ml^−1^ puromycin (Thermo Fisher Scientific, A1113803) for at least 2 weeks. Cultures with greater than 50% death rates after puromycin treatment were discarded.

### Fibroblast transdifferentiation

Transdifferentiation plates were prepared by coating plastic six-well or 15-cm plates with 10 μg ml^−1^ poly-d-lysine (PDL; Sigma, P6407-5MG) and 0.0001% (wt/vol) poly-l-ornithine (PLO; Sigma, P4957-50ML) for 24 h, followed by a 24 h incubation with 10 μg ml^−1^ laminin (Sigma, L2020-1MG). UNA fibroblasts were expanded to the desired cell number; in general, approximately threefold more surface area of fibroblasts was cultured for the desired plating surface of transdifferentiated neurons. For example, three UNA 15-cm plates (Genesee Scientific, 25-203; 435 cm^2^ total surface area) would seed one transdifferentiation 15-cm plate. Large fibroblast cultures (>10 million cells) were cultured in Corning CellBIND Surface HYPERFlask cell culture vessels (Sigma, CLS10034-24EA) following the manufacturer’s protocol. After the UNA fibroblasts were cultured to the desired cell number, fibroblasts were passaged to PDL-/PLO-/laminin-coated plates at 300% confluency for transdifferentiation. Transdifferentiating neurons were cultured for 3 weeks in NK (0.5× DMEM/F12, GlutaMAX supplement (Life, 10565042), 0.5× Neurobasal (Life, 21103049), 1× B-27 supplement (Life, 17504044), 1× N-2 supplement (Life, 17502048), 20 μg ml^−1^ laminin, 400 μg ml^−1^ dibutyryl-cAMP (db-cAMP; SelleckChem, S7858), 2 μg l^−1^ doxycycline (Sigma, D9881-10G), 5 μM dorsomorphin homolog 1 (ref. ^63^; Thermo Fisher Scientific, 412610), 0.5 μM LDN-193189 (Thermo Fisher Scientific, 50176042), 0.5 μM A83-01 (Stem Cell Technologies, 72022), 5 μM forskolin (Stem Cell Technologies, 72114), 3 μM CHIR-99021 (Thermo Fisher Scientific, 442310), 10 μM SB-431542 (Thermo Fisher Scientific, 161410) and 10 U ml^−1^ penicillin–streptomycin (Thermo Fisher Scientific, 15140122)) with media changes every 48 h. Cell morphology was checked every 48 h on a Zeiss Axio-Vert.A1 to ensure proper transdifferentiation progression. Mature transdifferentiated neurons were not fluorescently-sorted to ensure that there were enough cells for experiments with high input requirements like eCLIP and affinity pulldowns. For culturing longer than 3 weeks, media changes were reduced to once every 4 days.

### iPSC reprogramming and cell culture

Primary fibroblasts were reprogrammed using the CytoTune iPS 2.0 Sendai Kit (Thermo Fisher Scientific, A16517) at the titer recommended by the manufacturer. The fibroblasts were cultured in TFM for 1 week on an uncoated plate; afterward, the cells were passaged to plates coated with 1× Matrigel (Corning, 354277) and transitioned to ReproTeSR (Stem Cell Technologies, 05926) with media changes every 48 h. iPSC colonies were excised with a sterile needle and aspirated into a new plate for screening and validation. The iPSC cells were cultured on 1× Matrigel plates with mTeSR Plus (Stem Cell Technologies, 100-0276) media changes every 48 h; cells were passaged using Versene (Life, 15040066) dissociation reagent at a 1:5–10 split ratio. Long-term iPSC stocks were generated by resuspending the cells in 90% FBS and 10% DMSO.

### iPSC neuron differentiation

iPSCs were seeded on a 1× Matrigel-coated 12-well plate (Thermo Fisher Scientific, FB012928) at ~50% confluency. The iPSCs were transduced with the rtTA-Ngn2 lentivirus in mTeSR Plus without polybrene. After 48 h incubation at 37 °C with 5% CO_2_, the transduced iPSCs were treated with 1 μg ml^−1^ puromycin to select for iPSCs with doxycycline-inducible Ngn2 expression. The puromycin-resistant iPSCs were expanded to the desired cell number; in general, the iPSCs expanded fourfold once differentiation was initiated, so we seeded at ~25% confluency on 1× Matrigel-coated plates. Following seeding, the cells were cultured in mTeSR Plus with 2 μg ml^−1^ doxycycline for 72 h at 37 °C with 5% CO_2_. The cells were passaged to new 1× Matrigel-coated plates if needed and transitioned to NMM1 media (0.5× DMEM/F12, GlutaMAX supplement; 0.5× neurobasal; 1× B-27 supplement; 1× N-2 supplement; 2 μg ml^−1^ laminin; 0.5 mM db-cAMP; 20 ng μl^−1^ recombinant BDNF (Peprotech, 450-02-50UG); 20 ng μl^−1^ human GDNF (Peprotech, 450-10-50UG); 10 ng μl^−1^ human NT-3 (Peprotech, 450-03-50UG) and 10 U ml^−1^ penicillin–streptomycin) with half-media changes every 48 h. The cells were maintained in NMM1 for at least 3 weeks to ensure complete differentiation.

### *Mycoplasma* testing

Cell cultures were tested for the presence of *Mycoplasma* every month using the MycoAlert Mycoplasma Detection Kit (Lonza, LT07-318) following the manufacturer’s protocol. All new cell lines were quarantined and tested for *Mycoplasma* twice, at least 1 week apart, before being cultured with existing lines within the lab. The presence of *Mycoplasma* was also sporadically tested using DAPI staining and fluorescence microscopy (see below).

### Mouse models

Both male and female C57BL6/J mice (*Mus musculus*) used in this study were housed in the vivarium at the UCSD Moores Cancer Center or the Sanford Consortium for Regenerative Medicine in specific pathogen-free conditions. All protocols were approved by the UCSD Institutional Animal Care and Use Committee. Mice were killed at 1.5 months (young), 6 months (mid age) or 22–25 months (old) of age, and their brains were collected for immunohistochemistry and eCLIP (see below). Each experiment was performed with cohorts of two to four mice. The brains were excised from anesthetized mice, split laterally into the two hemispheres with each half going to our two experiments and immediately frozen at −80 °C (for eCLIP) or fixed (for immunohistochemistry). For fixation, mouse tissue was incubated in 4% paraformaldehyde at 4 °C for at least 24 h. The tissues were embedded in a cryomold in OCT solution (Sakura Finetek, 4583). Cryostat-sectioned tissue (20 μm) was mounted on Superfrost^+^ Slides (Thermo Fisher Scientific, 22037246) and stored at −80 °C until used for immunohistochemistry (see below).

### Human brain models

Control human brain tissue samples were obtained from the UCSD ALS repository, which is compliant with HIPAA informed consent procedures and approved by the institutional review board (CA, 120056). The brain tissue was dissected in an autopsy suite (usually with a short postmortem interval of less than 6 h) and either immediately frozen for biochemical studies or fixed in neutral buffered formalin for 2 weeks. Frontal cortex derived from three mid-age and three old-age patient samples (Supplementary Table 6) was used for this study. Human brain tissue is available via the Target ALS Multicenter Human Postmortem Tissue Core and can be requested at the following webpage: https://www.targetals.org/resource/human-postmortem-tissue-core/.

### Bisulfite sequencing

At least 1 × 10^5^ cells were pelleted and stored at −20 °C for genomic DNA extraction. Genomic DNA was purified from the cells using the DNeasy Blood & Tissue Kit (Qiagen, 69504) following the manufacturer’s protocol. Three micrograms of genomic DNA were used with the Infinium MethylationEPIC v2.0 Kit (Illumina, 20087706) at the UCSD IGM Genomics Center. Epic methylation data were submitted to the DNA Methylation Age Calculator maintained by the Clock Foundation (https://dnamage.clockfoundation.org/), which allowed us to approximate the age of cultured neurons^17^. We used the ‘Skin and Blood Aging Clock’ result from the Calculator output. Excel was used to plot the bisulfite aging data versus the actual age of the donor.

### MEA

Mature transdifferentiated neurons were seeded onto PDL-/PLO-/laminin-coated Cytoview MEA 48 plate (Axion Biosystems, M768-tMEA-48B). Approximately 1.5 × 105 neurons were seeded in a 50 μl droplet to concentrate the neurons on top of the electrode arrays. After a 2 h incubation at 37 °C with 5% CO_2_ to allow the cells to adhere, 150 µl additional media was added to each well. Cells were cultured for at least 48 h before MEA recordings.

MEA recordings were taken by a Maestro Classic MEA system (Axion Biosystems) using Axion Integrated Studio Acquisition software (v.2.1.5). Cultures were maintained at 37 °C with the addition of 10 mM HEPES (pH 7.5) to support CO_2_ buffering during recording. Neuronal activity was acquired at a sampling frequency of 12.5 kHz, using a Butterworth band-pass filter (200–3,000 Hz) and adaptive spike detector set at 5.5 s.d. from mean background noise. An electrode was defined as active with a mean firing rate greater than 0.017 Hz. Data acquisition for each condition consisted of a minimum of three 5-min recording sessions over the course of 30 min. Thresholded spike data (.spk files) were analyzed using Axion NeuralMetricTool software (v.2.1.5), evaluating weighted mean firing rate per well.

To measure induced action potentials, baseline neuronal activity was recorded for 30 min before the addition of 50 μM bicuculline (Tocris, 2503). Drug-induced neuronal activity was recorded following a 5-min equilibration period in the MEA system. Media was then refreshed, and neuronal activity was immediately recorded to assess recovery to induced activity (see ‘washout’ in Extended Data Fig. 1). GraphPad Prism 10 was used to plot the MEA data, and Prism also calculated statistical significance using Welch’s *t* test.

### Immunofluorescence

Ibidi μ-slide 18-well chambers (Ibidi, 81811) were coated as described above with PDL/PLO/laminin or 1× Matrigel for transdifferentiated and iPSC-derived neurons, respectively. Cells were transferred to the Ibidi μ-slides at least 24 h before immunofluorescence. Stress, recovery and inhibition treatments were applied at least 24 h after passaging to the Ibidi μ-slides—sodium arsenite (Thermo Fisher Scientific, 15654770) for 1 h at 0.5 mM; thapsigargin (Thermo Fisher Scientific, T7458) for 1 h at 1 μM; ganetespib (SelleckChem, S1159) for 24 h at 50 nM. To fix the cells, paraformaldehyde (Thermo Fisher Scientific, 50980492) was added to the cell media to a final concentration of 4% (vol/vol), which was then incubated at 4 °C with gentle rocking for 45 min. The samples were washed three times for 5 min each with 1× dPBS. Fixed cells were then permeabilized with 1× dPBS with 0.1% (vol/vol) Triton X-100 (Sigma, X100-500ML) and 5% (vol/vol) donkey serum (Jackson ImmunoResearch, 017-000-121) for 45 min at 25 °C. The permeabilized samples were washed three times for 1 min each with 1× dPBS with 0.1% (vol/vol) Triton X-100 (dPBST). Primary antibodies were added to antibody solution (1× dPBST with 0.1% (wt/vol) bovine serum albumin; Thermo Fisher Scientific, 10773877) at the concentrations listed in Supplementary Table 9 and incubated for 18 h with gentle rocking at 4 °C. The cells were then washed for 5 min three times with dPBST. Secondary antibodies were diluted in antibody solution (Supplementary Table 9) and incubated with the samples for 1 h with gentle rocking at 25 °C. Following this incubation, the cells were washed for 5 min three times with 1× dPBS, and Prolong Diamond Antifade Mountant with DAPI (Thermo Fisher Scientific, P36971) was used for long-term storage of the slides at 4 °C. The immunofluorescence samples were imaged on a Zeiss LSM-880 microscope with 405, 488, 561 and 633 nm lasers using a ×63/1.4 oil immersion objective. Confocal imaging data were acquired as single slices, and line averaging was used to improve signal-to-noise ratio. For Airyscan imaging data, processing of all channels was performed in Zen software using super-resolution settings.

Immunofluorescence images were processed with ImageJ for visualization and quantification. To determine the nuclear fraction of proteins, a nuclear mask was created and used to calculate the nuclear and cytoplasmic intensity independently. The nuclear intensity was divided by the total intensity to derive the nuclear fraction. For the intensity of the entire cell, the nuclear masking step was skipped. To determine the number and shape of stress granules, difference of Gaussians filtering was used to remove background noise and to enhance G3BP1 foci before applying an intensity auto threshold. Using these thresholded images, G3BP1 foci were masked as regions of interest and were validated manually—false foci were removed, and any foci that were missed by the automatic detection were added. The BioVoxxel Toolbox Extended Particle Analyzer (https://imagej.net/plugins/biovoxxel-toolbox) was used to calculate circularity and roundness of G3BP1 foci. The number of stress granules per cell was calculated by dividing the number of stress granules by the number of nuclei in each image. Data were visualized using GraphPad Prism 10, Welch’s *t* test was calculated in Excel and statistical outliers were discarded using Grubbs’s outlier test.

### Brain tissue immunohistochemistry

Formalin-fixed paraffin-embedded samples (that is, human brain tissue) were deparaffinized using two 5 min incubations in CitriSolv (Thermo Fisher Scientific, 04-355-121) at 25 °C, then washed twice in 100% (vol/vol) ethanol for 5 min each before proceeding to rehydration. Paraformaldehyde-treated samples (that is, mouse brain tissue) were thawed at 25 °C for 30 min before rehydration.

All tissue samples were first rehydrated using decreasing serial washes (95%, 85% and 75%) of ethanol at 25 °C and then a final wash in dH_2_O before moving immediately to antigen retrieval. All samples were treated with an antigen retrieval step to increase the antibody signal in the fixed tissue. In brief, 1× citrate buffer (Abcam, ab93678) was heated in a conventional microwave for 90 s. The rehydrated/deparaffinized slides were incubated in the heated 1× citrate buffer and incubated at 98 °C in a preheated laboratory oven for 30 min. The slides were cooled to 25 °C for 20 min, washed twice in 1× dPBS and used immediately for antibody staining. The tissue samples were surrounded by a hydrophobic barrier drawn with barrier pen (Thermo Fisher Scientific, NC9545623). To permeabilize the brain tissue, the samples were washed three times with SuperBlock (Thermo Fisher Scientific, 37515) for 5 min each and then incubated in SuperBlock for 45 min at 25 °C. After an additional three washes with SuperBlock, the samples were incubated in the primary antibody diluted in SuperBlock for 16 h at 4 °C; this step was repeated for the secondary antibody incubation, for which the sample was incubated at 25 °C for 6 h. The brains were then washed an additional three times with 1× dPBS, incubated with DAPI for 10 min, and mounted with Prolong Diamond. High magnification (×63) images were acquired on an LSM-880 microscope as described above. Tile scans were acquired on a Zeiss LSM-780 microscope at ×10 magnification. Map2^+^ neurons were manually identified and scored for cytoplasmic TDP-43 foci, and the results were plotted using GraphPad Prism 10. Statistics (Welch’s *t* test) were also calculated in Prism.

### Fluorescence in situ hybridization

The immunofluorescence protocol was followed through the secondary antibody staining step as described above. After washing the cells three times with 1× dPBS, the cells were fixed again with 4% (vol/vol) paraformaldehyde for an additional 10 min at 4 °C. Following fixation, the cells were washed with dPBST twice and incubated in FISH-W buffer (10% (vol/vol) formamide in 2× SSC) for 5 min at 25 °C. After the FISH-W treatment, the poly(A) probe was added to a final concentration of 125 nM in FISH-H buffer (2 mg ml^−1^ BSA + 10% (wt/vol) dextran sulfate in 2× SSC) and incubated for 2 h at 37 °C in a humidified chamber. The cells were then washed twice with FISH-W buffer and twice more with 2× SSC at 37 °C for 5 min; all washes were performed in the humidified chamber. The slides were then mounted with Prolong Diamond and imaged and analyzed as described in the Immunofluorescence.

### Western blot

At least 1 × 10^5^ cells were pelleted and stored at −80 °C or used immediately for western blotting. Cell pellets were resuspended in 200 μl RIPA lysis and extraction buffer (Thermo Fisher Scientific, 89900) supplemented with 2× Halt protease and phosphatase inhibitor cocktail (Thermo Fisher Scientific, 78440). The resuspended cells were incubated with rotation at 4 °C for 15 min, and they were further lysed by sonicating for 10 min (30 s/30 s off cycles). Lysed cells were centrifuged at 16,000*g* for 15 min at 4 °C. The supernatant fraction was transferred to a new tube and combined with 4× NuPAGE LDS sample buffer (Thermo Fisher Scientific, NP0007) supplemented with 100 mM β-mercaptoethanol (Sigma, M3148-250ML). The samples were then heated to 70 °C for 10 min and loaded onto ten-well NuPAGE 4–12% Bis–Tris Mini Protein Gels (Life, NP0335BOX). Gels were electrophoresed in the Novex XCell SureLock Mini-Cell Electrophoresis System (Thermo Fisher Scientific, EI0001) at 150 V for 75 min. The samples were then transferred using the dry iBlot system for most proteins (iBlot 2 Transfer Stacks; Life, IB24002). Once the samples were transferred to the PVDF membrane, the membranes were blocked in 5% (wt/vol) milk powder (Apex Bioresearch, 20-241) in 1× TBST (1× TBS (Thermo Fisher Scientific, AAJ62662K7) and 0.1% (vol/vol) Tween-20 (Sigma, P9416)) for 30 min at 25 °C. Primary antibodies were diluted to the concentrations listed in Supplementary Table 10 in 5% (wt/vol) milk powder in 1× TBST and incubated overnight with the blocked membrane at 4 °C with gentle rocking. The membrane was then washed thoroughly three times with 1× TBST for 5 min each wash at 25 °C. Secondary horseradish peroxidase (HRP)-conjugated antibodies were diluted 1:2,000 in 5% (wt/vol) milk powder in 1× TBST and incubated with the membrane for 1 h at 25 °C with gentle rocking. After an additional three washes with 1× TBST, the HRP-conjugated secondary antibodies were developed with ECL western blotting substrate (Life, 32106) and imaged on an Azure 600 Western Blot Imager (Azure Biosystems). The western blot images were analyzed using ImageJ to determine the intensity of individual bands, which were normalized to the loading control. The relative expression was plotted using GraphPad Prism 10, and Welch’s *t* test was used to calculate significance in Prism.

For phosphoproteins, a few modifications were made to the abovementioned protocol. First, β-mercaptoethanol was omitted from the LDS sample buffer. Second, a wet transfer in transfer buffer (1× NuPAGE transfer buffer (Life, NP0006) and 10% (vol/vol) methanol (Thermo Fisher Scientific, A411-4)) was performed for 16 h at 40 V using the Immobilon-FL PVDF membrane (Sigma, IPFL00010). Third, 5% (wt/vol) BSA in 1× TBST was used for blocking and for antibody incubations. Fourth, the Li-Cor IR dye-conjugated antibody was used for the secondary staining, and we imaged using the IR channels of the Azure 600 Western Blot Imager. For phospho-Westerns, normalization was performed to the total protein stain.

### RNA-seq

At least 1 × 10^5^ cells were pelleted for RNA-seq experiments. For long-term storage of samples before RNA-seq, the cells were stored in Trizol (Thermo Fisher Scientific, 15596026) at −20 °C and precipitated following the manufacturer’s protocol before RNA isolation and library preparation. RNA was isolated using the RNeasy Kit (Qiagen, 74104) following the manufacturer’s protocol, and libraries were prepared using the stranded mRNA prep kit (Illumina, 20040532). The IDT for Illumina Indices (Illumina, 20027213) was used for sequence barcoding, and sequencing was performed on a NextSeq 2000 using the appropriate flow cell attachment. RNA-seq data were trimmed with cutadapt 3.4 and aligned using STAR aligner 2.7.6a to the human genome (Gencode, hg38)^64^. From the STAR aligner output BAM files, subread featureCounts was used to quantify the number of reads in each gene^65^. Differential transcript expression was calculated using Salmon^66^ from FASTQ files using its pseudoalignment strategy. Alternative splicing was analyzed using rMATS 4.2.0 (ref. ^67^). RNA-seq data were visualized using R for differential expression and IGV for read density.

### Proteomics

At least 1 × 10^5^ cells were pelleted for whole-cell proteomics experiments. Cell pellets were resuspended in freshly made proteomics lysis buffer (8 M urea (Life, 15505-035), 75 mM NaCl (Thermo Fisher Scientific, AM9759), 50 mM Tris–HCl (pH 8.0; Thermo Fisher Scientific, 15568025), 1 mM NaF (Sigma, 201154-100G), 1 mM sodium orthovanadate (Sigma, S6508-10G) and 1 mM β-glycerophosphate (Sigma, G9422-10G)). The suspension was sonicated for 400 s (10 s on/30 s off cycles) and cleared by centrifugation at 16,000*g* for 10 min at 4 °C. Protein concentrations were measured using the Pierce BCA Protein Assay Kit (Thermo Fisher Scientific, 23225) following the manufacturer’s instructions. Approximately 20 μg of protein was reduced with 10 mM TCEP (Sigma, 4706) for 30 min, alkylated with 15 mM freshly prepared iodoacetamide (IAA; Sigma, I6125-10G) for 45 min in the dark, and then treated with 10 mM DTT for 15 min in the dark. The samples were diluted to a final volume of 400 μl with 50 mM Tris–HCl (pH 8.0) to achieve a urea concentration of less than 1 M and maintain a neutral pH. For digestion, 200 ng LysC endoprotease (NEB, P8109S) and 400 ng trypsin (NEB, P8101S) were added to the samples, which were then incubated at 37 °C with shaking at 300 rpm for 16 h. The digestion was terminated by adding formic acid (Sigma, F0507-100ML) to achieve a final concentration of 1%.

To prepare human brain tissue for proteomics, 10 mg of brain tissue was dissected and added to freshly made tissue lysis buffer (8 M urea (Life, 15505-035), 50 mM NaCl (Thermo Fisher Scientific, AM9759), 50 mM Tris–HCl (pH 8.0; Thermo Fisher Scientific, 15568025), 4% SDS (Thermo Fisher Scientific, 24730020) and protease inhibitor cocktail (Millipore, 539134-1SET)). After a 10-min incubation on ice, the tissue was heated at 95 °C for 10 min, sonicated for 400 s (10 s on/30 s off cycles) and cleared by centrifugation at 16,000*g* for 10 min at 4 °C. Protein concentrations were measured using the Pierce BCA Protein Assay Kit (Thermo Fisher Scientific, 23225) following the manufacturer’s instructions. In total, 20 μg of protein was reduced with 10 mM TCEP (Sigma, 4706) for 30 min, alkylated with 15 mM freshly prepared IAA (Sigma, I6125) for 45 min in the dark and then treated with 10 mM DTT for 15 min in the dark. The protein was purified via methanol–chloroform precipitation and solubilized in 100 mM TEAB (Sigma, T7408). For digestion, 200 ng LysC endoprotease (NEB, P8109S) and 400 ng trypsin (NEB, P8101S) were added to the samples, which were then incubated at 37 °C with shaking at 300 rpm for 16 h. The digestion was terminated by adding formic acid (Sigma, F0507) to a final concentration of 1%.

The digested peptides for all samples were desalted using the Stage-Tip method and then analyzed by a liquid chromatography (LC)–MS/MS setup. The setup included a timsTOF Pro 2 mass spectrometer (Bruker Daltonics) coupled to a nanoElute 2 nano-LC system (Bruker Daltonics), using a CaptiveSpray ion source. The peptides were eluted through a reversed-phase C18 column (PepSep, 25 cm × 150 µm, 1.5 µm) maintained at 50 °C over a 50 min gradient of 5–35% solvent B (acetonitrile in 0.1% formic acid) at a flow rate of 500 nl min^−1^. The peptides were analyzed using data-independent acquisition (dia) parallel accumulation-serial fragmentation (PASEF) mode. The isolation windows for diaPASEF were determined based on data-dependent acquisition (dda)-PASEF data^68^. The settings for ddaPASEF included the following: a ramp time and an accumulation time of 75 ms each, one MS scan and ten PASEF MS2 ramps per acquisition cycle. The MS survey scan covered a mass-to-charge (*m*/*z*) ratio range of 100–1,700 *m*/*z* and ion mobility (1/k0) range of 0.6–1.6 V s cm^−2^. Precursors with up to five charges were selected, with an active exclusion time of 0.4 min. The quadrupole isolation width was set at 2 *m*/*z* for *m*/*z* values below 700 and 3 *m*/*z* for *m*/*z* values above 800, with linear interpolation for *m*/*z* values of 700–800. For diaPASEF, the isolation windows were designed to encompass the precursor distribution across the *m*/*z*-1/k0 plane as defined by the ddaPASEF data, ranging from 323.6 to 1221.6 *m*/*z* and 0.7 to 1.34 V s cm^−2^ in 1/k0. Each 75 ms diaPASEF scan spanned a 40 Da mass width with a 1 Da mass overlap, and the number of windows varied to cover the entire ion mobility spectrum (creating 23 windows for ten diaPASEF scan cycles)^69^.

The diaPASEF raw files were processed using a library-free search approach in DIA-NN (v.1.8.1)^70^. An in silico digestion of a UniProt reviewed *Homo sapiens* database (December 2022) was used to predict the spectral library. The search parameters applied included the following: tryptic digestion with up to two missed cleavages, carbamidomethylation of cysteine as static modification, oxidation of methionine and N-terminal acetylation as variable modifications. The precursor false discovery rate threshold was set at 1%. The quantification strategy for precursors was set to robust LC (high precision), and cross-run normalization was set to retention time-dependent. Match between run enabled for the protein group matrix file generated by DIA-NN, filtered at 1% false discovery rate using global *q* values for protein groups and global and run-specific *q* values for precursors, was used for downstream and statistical analysis. The statistical analysis was performed using the limma package within the R framework^71^. Proteomics data were visualized using R and Excel.

### eCLIP

At least 1 × 10^7^ cells were used for eCLIP experiments; for transdifferentiated neurons, this usually required pooling three 15-cm plates into a single pellet, whereas other cell types required no more than one 15-cm plate. Live cells were washed with 1× dPBS before cross-linking. The cells were exposed to 4,000 μJ 254 nm UV light using a UVP CL-1000 Ultraviolet Cross-linker and pooled as needed for pelleting. Cross-linked pellets were flash-frozen and stored at −80 °C until the rest of the experiment was performed.

eCLIP was performed essentially as described previously^30,72^. Immunoprecipitation-validated antibodies (Supplementary Table 11) were reacted with M-280 sheep anti-rabbit IgG Dynabeads (Life, 11204D). Cross-linked pellets were resuspended in 1 ml 1× eCLIP lysis buffer (50 mM Tris–HCl (pH 7.4), 100 mM NaCl, 1% (vol/vol) IGEPAL-CA630 (Sigma, I8896); 0.1% (vol/vol) SDS (Thermo Fisher Scientific, 24730020), 0.5% (wt/vol) sodium deoxycholate (Sigma, D6750)) supplemented with fresh protease inhibitor cocktail (Millipore, 539134-1SET). After a 5-min incubation at 4 °C, the cells were sonicated for 5 min (30 s on/30 s off cycles). Following sonication, 5 U RNase I (Life, AM2295) and 10 U DNase (Life, AM2239) were added to the lysed cells, which were then incubated at 37 °C for 5 min at 1200 rpm. The RNase digestion was stopped by immediately cooling the samples to 4 °C and adding 440 U Murine RNase Inhibitor (NEB, M0314L). Cell debris was pelleted by centrifuging at 15,000*g* for 3 min at 4 °C. The supernatant fraction was transferred to a new tube, and the Dynabead-conjugated antibodies were added to the cell lysate. The immunoprecipitation reaction was incubated for 16 h at 4 °C with rotation.

Following immunoprecipitation, 40 μl was removed from the whole reaction for the input sample, which was stored at 4 °C until gel loading. The Dynabeads were washed twice with eCLIP HSW buffer (50 mM Tris–HCl (pH 7.4), 1 M NaCl, 1 mM EDTA (pH 8.0), 1% (vol/vol) IGEPAL-CA630, 0.1% (vol/vol) SDS and 0.5% (wt/vol) sodium deoxycholate) and three times with eCLIP NSW buffer (20 mM Tris–HCl (pH 7.4), 10 mM MgCl_2_, 0.2% (vol/vol) Tween-20 and 5 mM NaCl) using a DynaMag-2 (Thermo Fisher Scientific, 12321D) magnet. After washing, the dry beads were resuspended in 1× fast AP buffer supplemented with 3 U FastAP (Life, EF0652), 4 U DNase and 80 U Murine RNase Inhibitor. Samples were incubated at 37 °C for 10 min at 1,200 rpm. The samples were then diluted in 1× PNK7 buffer with 40 U T4 PNK (NEB, M0201L) and incubated at 37 °C for a further 20 min at 1,200 rpm. The beads were washed with HSW buffer twice and NSW buffer three times. The dry beads were resuspended in eCLIP 3′ RNA ligation buffer (1.2× RNA ligase buffer, 1.2 μM ATP, 3.6% (vol/vol) DMSO, 0.024% (vol/vol) Tween-20 and 50% (wt/vol) PEG 8000) supplemented with 72 U T4 RNA ligase 1 (NEB, M0437M), 16 U Murine RNase Inhibitor and 4 μM 3′ IP RNA Adaptor sequence (/5Phos/rNrNrGrArUrArUrCrGrArArGrArUrCrGrGrArArGrArGrCrArCrArCrGrUrC/3SpC3/), and the ligation reaction was incubated at 25 °C for 75 min with rotation. Following ligation, the beads were washed with NSW buffer twice, HSW buffer thrice and NSW buffer thrice. Samples were prepared for loading by adding NSW buffer and removing 20% of the IP sample for loading onto a western blot for visualization. The remaining 80% of the IP sample was used for the remainder of the eCLIP protocol. IP and input samples were mixed with 4× NuPAGE LDS buffer and 100 mM DTT, heated to 70 °C for 10 min, magnetically separated and loaded onto 4–12% Bis–Tris gels. The samples were electrophoresed for 75 min at 150 V using the Novex XCell SureLock Mini-Cell Electrophoresis System as described above for western blots. The RNA-containing gels were transferred using a wet transfer system to Amersham Protran Nitrocellulose Western blotting membranes (Sigma, GE10600008) for 16 h at 30 V. The regions corresponding to the molecular weight of the protein visualized on the control western blot (+75 kDa to account for RNA binding) were excised using clean razor blades and transferred to sterile tubes. RNA was released from the membrane by resuspending in eCLIP PKS buffer (100 mM Tris–HCl (pH 7.4), 50 mM NaCl, 10 mM EDTA and 0.2% (vol/vol) SDS) supplemented with 16 U proteinase K (NEB, P8107S). The samples were incubated at 37 °C for 20 min at 1,200 rpm and then further incubated at 50 °C for 20 min at 1,200 rpm. The released RNA was further cleaned using the RNA Clean & Concentrator Kit (Zymo, R1016) following the manufacturer’s protocol.

The input RNA samples were treated with FastAP and T4 PNK as described for IP samples above, and the RNA was isolated using the RNA Clean & Concentrator Kit. The input sample was annealed to 1 μM 3′ Input RNA Adaptor sequence (/5Phos/rArGrArUrCrGrGrArArGrArGrCrArCrArCrGrUrC/3SpC3/) at 65 °C for 2 min, and it was then ligated using the eCLIP 3′ RNA ligase buffer conditions mentioned above. The ligated input RNA was further cleaned using MyONE Silane Beads (Life, 37002D) that were equilibrated with RLT buffer (Qiagen, 79216) supplemented with 0.025% Tween-20. The RNA sample was added to 3.0 volumes of 100% ethanol and reacted with the beads for 10 min with trituration. The beads were then magnetically separated, washed three times with 80% (vol/vol) ethanol, air-dried for 5 min and eluted with dH_2_O.

Input and IP RNA were then reverse transcribed to cDNA by annealing 3 μM reverse transcriptase primer mix (/5Phos/NNNNNNNNNNAGATCGGAAGAGCGTCGTGT/3SpC3/) at 65 °C for 2 min. The annealed RNA was added to 1× first-strand buffer with 5 mM DTT, 8 U Murine RNase Inhibitor and 120 U Superscript III Enzyme (Life, 18080044) and incubated at 55 °C for 20 min. The cDNA was incubated with 2.5 μl ExoSAP-IT (Thermo Fisher Scientific, 78201.1.1ML) for 15 min at 37 °C. EDTA (10 mM) was used to stop the cleanup reaction, and RNA was removed by incubating with 100 mM NaOH at 70 °C for 10 min. The solution was neutralized by adding 100 mM HCl. The cDNA was cleaned up using the MyONE Silane Beads as described above, but the samples were not eluted from the beads. Instead, the beads were resuspended in 0.5 μM 5′ cDNA Adaptor Sequence (CAGACGTGTGCTCTTCCGA) and annealed at 70 °C for 2 min. The sample was then ligated for 16 h in eCLIP cDNA ligation buffer (10 mM Tris–HCl (pH 7.4), 0.1 mM EDTA, 0.01% (vol/vol) Tween-20 and 7.77 (vol/vol) DMSO) with rotation. Ligated cDNAs were further incubated with fresh MyONE Silane Beads as described above. The eluted cDNA was quantified by qPCR and PCR-amplified for sequencing with Illumina adaptors. Sequencing data were analyzed using the Skipper pipeline that was previously described^73^, which maps sequencing peaks to the human genome (hg38) using the input sample as background. The Skipper pipeline calculated the enriched RNA-binding windows and HOMER binding motifs; enriched eCLIP binding windows were further analyzed and visualized using R or Excel.

Tissue eCLIP was performed essentially as described above except for a few changes, which are as follows: (1) mouse and human brain tissue was homogenized in 1× dPBS and plated onto 10-cm plates, (2) tissue was irradiated with two 4,000 μJ cross-linking cycles, (3) brain tissue in 1× dPBS was lysed by diluting with 5× eCLIP lysis buffer, (4) the mouse eCLIP reads were mapped to the mouse genome (mm10) instead of the human genome and (5) cohorts of two to four mice and three human donors were used for each age group/cohort in Figs. 2 and 6, respectively.

### AP-MS

At least 5 × 10^6^ cells were pelleted for AP-MS. Cell pellets were resuspended in IP lysis buffer (50 mM Tris–HCl (pH 7.4), 150 mM NaCl, 1 mM EDTA (pH 8.0) and 0.5% (vol/vol) IGEPAL-CA630) supplemented with fresh protease inhibitor cocktail. The cells were then sonicated for 5 min (30 s on/30 s off), and the lysate was centrifuged at 14,000*g* for 10 min at 4 °C. Following centrifugation, the supernatant fraction was transferred to a new tube, and the protein content of each sample was estimated using the Pierce BCA Protein Assay Kit. Approximately 3 mg of protein was combined with Dynabead-conjugated antibodies (see above; Supplementary Table 11) and incubated for 16 h at 4 °C with rotation. The beads were then washed three times with IP lysis buffer and three times with IP wash buffer (50 mM Tris–HCl (pH 7.4) and 150 mM NaCl). Any residual liquid in the tube was removed, and the dried beads were submitted to the Sanford Burnham Prebys Proteomics Core for MS. The MS for each protein was averaged across sample replicates, and the log_2_(fold change) and −log_10_(*P*) were calculated for the proteins. Proteins corresponding to the Kyoto Encyclopedia of Genes and Genomes (KEGG) terms in various relevant pathways were highlighted in the relevant plots.

### Tandem ubiquitin binding entities (TUBE) pulldown MS

Magnetic TUBE beads (LifeSensors, UM402M) were prepared for the pulldown by combining 300 μl bead slurry per sample and washing three times with TUBE IP buffer (50 mM Tris–HCl (pH 8), 150 mM NaCl and 0.1% IGEPAL-CA630) on a magnetic rack. The washed beads were resuspended in 300 μl TUBE IP buffer per sample. At least 5 × 10^6^ cells were pelleted for the TUBE pulldown. Cells were resuspended in TUBE lysis buffer (50 mM Tris–HCl (pH 8), 150 mM NaCl, 1% IGEPAL-CA630, 50 mM freshly made N-ethylmaleimide, 50 mM NaF and 10 mM sodium β-glycerophosphate) supplemented with fresh protease inhibitor and rotated end-over-end for 15 min at 4 °C. After rotation, the cells were sonicated for 5 min (30 s on/30 s off), and the lysate was centrifuged at 15,000*g* for 10 min at 4 °C. The protein content was estimated using the Pierce BCA Protein Assay Kit, and 3 mg of protein was combined with 300 μl of the washed magnetic TUBE beads from above. The lysate was incubated with the beads using end-over-end rotation for 18 h at 4 °C. Following this incubation, the beads were isolated using a magnetic rack and washed three times with TUBE IP buffer and then three times with IP wash buffer. The washed beads were resuspended in 100 μl (50 mM) Tris (pH 8). The peptides were reduced and digested using TCEP, IAA, DTT, trypsin and LysC as described above in the ‘Proteomics’ section. The proteases were neutralized with 1% formic acid. MS was performed essentially as described above in the ‘Proteomics’ section with one modification—the samples were searched using a diglycine remnant-containing peptide library provided by Eric Fischer (Dana-Farber Cancer Institute).

### Ribo-seq

For Ribo-seq, we simultaneously generated footprinting and bulk RNA-seq samples so that they were processed in the same manner. Samples were collected as previously described^74^. Briefly, cells were cultured in 15 cm plates. Cells were treated with 100 μg ml^−1^ cycloheximide for 1 min at 37 °C followed by two washes with ice-cold 1× dPBS supplemented with 100 μg ml^−1^ cycloheximide. Ribo-seq lysis buffer (12.5 mM Tris (pH 7.0), 7.5 mM Tris (pH 8.0), 150 mM NaCl, 5 mM MgCl_2_, 100 μg ml^−1^ cycloheximide, 1 mM DTT, 1% (vol/vol) Triton X-100 and 20 U ml DNase) was added to the washed cells, which were then collected and centrifuged at 16,000*g* for 10 min at 4 °C.

The cell lysate was treated with 250 U RNase I (Thermo Fisher Scientific, AM2295) at 25 °C for 45 min. The RNase digestion was halted by adding 200 U SUPERase Inhibitor (Thermo Fisher Scientific, AM2694) and transferring to 4 °C. Samples were loaded onto a sucrose gradient (34% sucrose, 20 mM Tris (pH 7.5), 150 mM NaCl, 5 mM MgCl_2_, 1 mM dithiothreitol and 100 μg ml^−1^ cycloheximide) and centrifuged for 1 h at 300,000*g* in a TLA-110 rotor (Beckman Coulter, 366735) at 4 °C. Following centrifugation, the polysome pellet was resuspended in 1 ml TRIzol (Thermo Fisher Scientific, 15596026), and RNA was extracted following the manufacturer’s protocol by chloroform-based separation. Ten micrograms of total RNA was used for the extraction of 28–34-nucleotide mRNA fragments that were resolved with a 15% UREA-TBE gel for ribosomal footprints. Ribo-seq libraries were then processed as described^74,75^.

Corresponding samples were grown in parallel for RNA-seq in six-well plates and treated as described previously^75^. For RNA-seq, cells were collected with Tri-Reagent (Ambion, AM9738) for RNA extraction and used for polyA selection with Dynabeads mRNA DIRECT Purification Kit (Invitrogen, 61012). Then, additional DNA degradation and 3′ mRNA resolution and phosphorylation were carried out by DNaseI and 3′ dephosphorylation using FastAP Thermosensitive Alkaline Phosphatase (Thermo Fisher Scientific, EF0651) and T4 PNK (NEB, M0201S), followed by 3′ adaptor ligation using T4 ligase (NEB, M0437M). The products were used for reverse transcription with SuperScript III (Invitrogen, 18080093) for first-strand cDNA synthesis. The cDNA products were 3′ ligated with a second adaptor using T4 ligase and amplification with 8–12 cycles of PCR for final library products of 200–300 bp.

Sequencing reads for Ribo-seq and corresponding RNA-seq samples were aligned as previously described^76^. In brief, linker (CTGTAGGCACCATCAAT) and poly(A) sequences were removed, and the remaining reads were aligned to rRNA. Unaligned reads were used for the alignment to the GRCh38 human sequence. Alignment was performed using Bowtie (v.1.1.229) with a maximum of two mismatches per read. Reads that were not aligned to the genome were aligned to sequences that spanned splice junctions. The aligned position on the genome was determined as the 5′ position of RNA-seq reads. For Ribo-seq reads, the ribosome P site was calculated using the 5′ end of the reads of the canonical ORFs with +12 for reads that were 28–29 bp and +13 for reads that were 30–33 bp.

### Statistics and reproducibility

No statistical method was used to predetermine sample sizes, and experiments were generally not anonymized or randomized during analysis. Outlier data points were only excluded if they tested as a significant outlier using Grubb’s test. For simple statistical comparisons, a two-tailed Welch’s *t* test (that is, a *t* test assuming a normal distribution but with unequal variances) was performed in most cases, and the degrees of freedom for significance scoring were approximated using Satterthwaite’s correction. We did not confirm normal distributions for each data distribution, but all individual data points are plotted for every experiment. Certain data analysis pipelines (for example, clusterprofiler for KEGG term enrichment or RMATs for splicing analysis) have their own statistical calculations built into the software, and although we note which test was used, the original work will provide greater detail about the statistical power of their calculations. For imaging data and blots, we performed each experiment at least in triplicate; certain imaging experiments (for example, Map2/Tubβ3/G3BP1 staining) were performed over ten times, and we observed strong reproducibility across many different biological and technical replicates. The figures shown in the manuscript are a representative sample of each experiment (Figs. 1b,c,e,f, 2a,c,j, 3b,c, 4a–c,h, 5a,c and 6d).

### Reporting summary

Further information on research design is available in the Nature Portfolio Reporting Summary linked to this article.

## Online content

Any methods, additional references, Nature Portfolio reporting summaries, source data, extended data, supplementary information, acknowledgements, peer review information; details of author contributions and competing interests; and statements of data and code availability are available at 10.1038/s41593-025-01952-z.

## Supplementary information

Supplementary Information Supplementary Tables 1–11.

Reporting Summary

## Source data

Source Data Fig. 1 Unprocessed p16INK4A western blot.

Source Data Fig. 3 Unprocessed eIF2α western blot.

Source Data Extended Data Fig. 10 Unprocessed eIF2α western blot.

## Data Availability

Raw and processed data are available at the following biorepositories: RNA-seq at NIH Gene Expression Omnibus (GEO; GSE276986); eCLIP-seq at NIH GEO (GSE276985); Ribo-seq at NIH GEO (GSE277082); whole-cell neuronal MS at PRIDE (PXD055825); TDP-43/G3BP1/HSP90α affinity pulldown and MS at PRIDE (PXD055904); TUBE AP-MS at PRIDE (PXD055826); human brain tissue MS at PRIDE (PXD055900); and imaging at Mendeley Data (10.17632/k497wp2x67.1)^77^. The human genome (hg38) and mouse genome (mm10) are available at the National Center for Biotechnology Information—hg38, GCF_000001405.26; mm10, GCF_000001635.20. Source data are provided with this paper.

## Code availability

No new code or software was created for this manuscript.
