# Supplementary material for: Neuronal aging causes mislocalization of splicing proteins and unchecked cellular stress
Source: Nat Neurosci. 2025 Jun 2;28(6):1174–84. doi: 10.1038/s41593-025-01952-z (PMC12148940; doi:10.1038/s41593-025-01952-z)
Supplement: Supplementary file 1 — Supplementary Tables 1–11. [file 41593_2025_1952_MOESM1_ESM.pdf]

nature neuroscience

Supplementary information https://doi.org/10.1038/s41593-025-01952-z

Neuronal aging causes mislocalization of splicing proteins and unchecked cellular stress

In the format provided by the authors and unedited

Supplementary Information

Supplementary Table 1: Significantly upregulated RNA-Seq KEGG pathways in aged neurons.

KEGG Pathway Name	KEGG Pathway ID	Enrichment Score	$-Log_{10}$(p-value)
Oxidative phosphorylation	hsa00190	0.8000	6.1944
Rheumatoid arthritis	hsa05323	0.7966	4.6745
Cytokine-cytokine receptor interaction	hsa04060	0.6792	6.6530
Lysosome	hsa04142	0.7172	4.1172
Protein processing in endoplasmic reticulum	hsa04141	0.6718	3.9226

Supplementary Table 2: Significantly up- and downregulated proteomics KEGG pathways in aged neurons.

KEGG Pathway Name[1]	KEGG Pathway ID	Enrichment Score	$-Log_{10}$(p-value)[2]
Spliceosome	hsa03040	-0.59857	8.65267
Parkinson's disease	hsa05012	0.78168	8.65267
Oxidative phosphorylation	hsa00190	0.76920	8.65267
DNA replication	hsa03030	-0.70288	5.75206
Alzheimer's disease	hsa05010	0.70784	8.65267
mRNA surveillance pathway	hsa03015	-0.56836	7.39168
RNA transport	hsa03013	-0.51501	8.65267
Huntington's disease	hsa05016	0.66860	8.65267
Cell cycle	hsa04110	-0.51974	6.61542
Protein digestion and absorption	hsa04974	0.81495	8.65267
Basal transcription factors	hsa03022	-0.63114	3.55550
Lysosome	hsa04142	0.64901	8.65267
N-Glycan biosynthesis	hsa00510	0.71395	6.43577
Citrate cycle (TCA cycle)	hsa00020	0.75486	6.16327
Adherens junction	hsa04520	-0.49078	3.93498
Ribosome biogenesis in eukaryotes	hsa03008	-0.47074	4.68836
Glycosaminoglycan degradation	hsa00531	0.85917	3.76482
Other glycan degradation	hsa00511	0.84073	3.40038
Fatty acid metabolism	hsa00071	0.67202	3.66429
Valine, leucine and isoleucine degradation	hsa00280	0.61288	3.72373
Antigen processing and presentation	hsa04612	0.66604	3.40038
Tight junction	hsa04530	-0.41539	3.46958
Nicotinate and nicotinamide metabolism	hsa00760	0.75137	3.05261
Protein processing in endoplasmic reticulum	hsa04141	0.51660	5.47702

[1] Italicized KEGG pathways are related to RNA metabolism.

[2] P-values were calculated by performing a hypergeometric test.

Supplementary Table 3: Splicing and stress granule proteins are segregated in transdifferentiated neurons.

	Protein[1]	Proteomics	TDP-43 Pulldown[2]		G3BP1 Pulldown[2]	
		$Log_2(FC)$	$Log_2(FC)$	$-Log_{10}$(p-value)[3]	$Log_2(FC)$	$-Log_{10}$(p-value)[3]
Stress Granules (hsa03019+10146)	PABPC1	0.2564	N.D.	N.D.	-0.7039	0.2201
	PABPC5	-0.0467	N.D.	N.D.	0.0202	0.0030
	PABPC4	0.0067	0.4711	0.4042	-0.1358	0.0292
	STAU2	-0.8915	-1.3283	1.2762	-2.0041	0.8221
	STAU1	-0.7897	-0.9024	0.1406	-2.5813	0.9962
	FMR1	-0.3084	-0.1468	0.1062	-0.7603	0.2128
	FXR1	-0.9243	-2.0526	0.5600	-2.2235	0.9130
	FXR2	-0.4239	-2.8064	0.6899	-1.3063	0.5139
	ELAVL1	-1.3755	0.8637	0.5910	-0.2742	0.0621
	G3BP1	-0.4272	N.D.	0.3010	-0.4022	0.2245
	G3BP2	-0.9055	-4.7910	0.4250	-1.3408	0.5661
	EIF4A3	-0.7261	-1.0063	0.2303	-3.7787	1.3190
	EIF4G1	0.1315	-1.1778	0.2235	-1.3467	0.4684
	CAPRIN1	-0.1291	-1.0373	0.5164	-0.3602	0.1350
Spliceosome (hsa03040)	SNRPD1	-1.0153	-4.7947	0.8318	-1.1679	0.5267
	SNRPD2	-0.5697	2.3624	1.0812	-1.3003	0.5700
	SNRPD3	-0.7595	-1.0047	0.2442	-2.9221	2.0015
	SNRPE	-0.2485	-0.7580	0.2107	-2.0008	0.6889
	SNRPF	-0.9616	1.6038	3.0458	-1.6949	0.8673
	SNRNP70	-0.6706	4.1985	0.9171	-3.1584	0.6770
	SNRPA	0.0196	N.D.	0.3010	-2.7215	1.0580
	RBM25	-0.8998	0.8592	1.0270	N.D.	0.3010
	DDX5	-0.9413	-3.5253	0.9651	-0.9779	0.4705
	SF3B2	-0.7756	3.9900	1.7179	N.D.	0.6840
	SF3B4	-0.1669	N.D.	1.7982	-2.7774	1.0928
	SF3B3	-0.6533	0.5396	0.2442	-3.4282	1.5735
	SF3B1	-0.5737	2.3130	0.8401	-3.1870	0.6926
	SF3B6	-0.5921	-1.3652	0.9342	N.D.	N.D.
	U2AF1	-0.7043	-0.7649	0.4207	-4.4605	0.9703
	U2AF2	-0.7625	-1.8983	0.9331	-3.9981	0.9590
	PUF60	-0.3891	2.4634	0.7399	N.D.	0.8556
	DHX15	-0.5844	-0.4705	0.7800	-4.1435	1.1193
	EFTUD2	-0.8873	2.0270	0.4698	N.D.	0.9120
	SNRNP200	-0.6674	2.6646	2.2583	-5.9556	0.5661
	PRPF8	-0.3964	4.5345	1.5504	-5.4999	0.8353
	DDX23	-0.4021	3.2147	1.5420	N.D.	0.3010
	PRPF38b	-1.0705	2.1290	1.4227	N.D.	N.D.
	PRPF19	-1.0179	1.9451	0.6872	-6.1665	2.8295
	HSPA8	0.1423	-3.9721	1.2322	-1.3359	0.6320
	PPIL1	-0.6990	0.6808	0.1832	N.D.	N.D.
	ACIN1	-1.4188	2.2484	0.8102	-3.3277	2.1791
	EIF4A3	-0.7261	-1.0063	0.2303	-3.7787	1.3190
	RBM8A	-0.7669	5.2253	0.7855	-2.5485	0.6389
	NCBP2	-0.4777	N.D.	0.4806	-0.0229	0.0043
	NCBP1	-0.6338	N.D.	0.9726	-0.2404	0.0523
	HNRNPA3	-0.7035	-0.9063	0.7457	-0.3256	0.1292
	RBMX	-1.5281	N.D.	N.D.	-2.4745	0.7807
	HNRNPA1	-1.6349	-4.4818	0.7796	-8.3727	0.8147
	HNRNPK	-0.9329	-0.4486	0.5958	-1.4321	0.5325
	HNRNPU	-0.7175	-2.4075	0.9256	-2.0342	0.7325
	HNRNPM	-1.2655	-2.9495	2.3118	-2.6483	0.7026

PCBP1	-0.4668	-3.4979	0.8571	-3.2856	1.0420
SRSF1	-0.9694	2.6130	0.6330	-0.7937	0.2601
SRSF2	-1.0997	2.1963	0.6685	-3.3710	0.5546
SRSF3	-1.0155	2.5224	1.0958	-1.5853	0.6021
SRSF4	-0.3301	5.8704	0.4355	-1.0612	0.5513
SRSF5	-0.9728	N.D.	0.8906	-4.5035	0.8356
SRSF6	-0.6660	1.1277	0.4263	-0.7956	0.2666
SRSF7	-0.9789	2.7014	0.8052	-1.8215	0.7018
SRSF9	-1.6674	4.4123	0.8983	-3.4140	1.7826
SRSF10	-1.1951	6.8295	0.7260	-1.5442	0.6363
TRA2A	-0.5616	3.7334	1.3688	-3.0959	0.9773
TRA2B	-0.6704	2.7104	1.3269	-1.7165	0.7761
HNRNPF	-0.6145	2.2503	0.8770	-1.5572	0.5401
HNRNPH1	-1.3568	-0.3604	1.3818	-1.4337	0.5691
PTBP1	-0.9330	1.0955	1.0440	-0.6839	0.1735
TARDBP[4]	-0.7237	0.3901	0.3113	-5.1397	1.1772

[1] Italicized proteins are highlighted in Figures 2 and 3.

[2] Proteins that were not detected in both samples are labeled N.D. (not detected). However, these proteins may have a p-value if the protein was detected in one sample but not the other sample, e.g. iPSC-derived neurons but not the transdifferentiated neurons. This is because a zero value would allow the calculation of a p-value even though a fold change cannot be calculated. These values were not plotted in Figures 2 or 3.

[3] P-values were calculated using a two-tailed Welch's t-test.

[4] TARDBP (TDP-43) was manually added to the spliceosome pathway due to its documented roles in splicing.

Supplementary Table 4: HSP90 chaperones are depleted from stress granules and the spliceosome in aged neurons.

	**Protein**[1]	**Proteomics**	**TDP-43 Pulldown**[2]		**G3BP1 Pulldown**[2]	
		Log_2(FC)	Log_2(FC)	$-Log_{10}$(p-value)[3]	Log_2(FC)	$-Log_{10}$(p-value)[3]
HSP90	*HSP90AA1*[4]	-0.3657	-5.6835	1.7640	-5.5749	2.2104
	HSP90AB1	-1.1077	-6.1818	1.1270	-7.0356	0.7878
	HSP90AB4	N.D.	N.D.	0.3010	N.D.	N.D.
	HSP90B1	1.7257	-0.3027	0.1357	-5.4020	0.4418

[1] Italicized proteins are highlighted in Figures 2 and 3.

[2] Proteins that were not detected in both samples are labeled N.D. (not detected). However, these proteins may have a p-value if the protein was detected in one sample but not the other sample, e.g. iPSC-derived neurons but not the transdifferentiated neurons. This is because a zero value would allow the calculation of a p-value even though a fold change cannot be calculated. These values were not plotted in Figures 2 or 3.

[3] P-values were calculated using a two-tailed Welch's t-test.

[4] HSP90AA1 was denoted as HSP90α throughout the manuscript.

Supplementary Table 5: HSP90α targets in oxidative phosphorylation have poor turnover rates.

	Protein[1]	RNA-Seq	Proteomics	HSP90α Pulldown[2]	
		$Log_2(FC)$	$Log_2(FC)$	$Log_2(FC)$	$-Log_{10}$(p-value)[3]
Oxidative Phosphorylation (hsa00190)	*CYCS*	1.1306	2.4126	1.4676	0.8947
	COX4I1	0.6576	2.9038	-1.2839	0.1785
	COX5B	0.7406	3.0528	-1.0803	0.3255
	COX6B1	0.5553	1.4682	N.D.	0.3010
	COX6C	1.3631	2.3986	0.4673	0.0816
	COX7A2	0.5462	2.9153	N.D.	0.3010
	CYC1	0.4474	2.4264	N.D.	0.3010
	UQCRQ	2.1802	2.0778	N.D.	0.3010
	UQCR10	0.9740	2.8174	N.D.	0.3010
	MT-ATP6	1.8138	3.1949	N.D.	0.6405
	MT-ATP8	1.5546	2.6165	N.D.	0.3010
	MT-CO2	2.3328	3.2582	-0.7483	0.3384
	NDUFA4	0.5922	2.3132	N.D.	0.3010
	NDUFA5	0.2180	1.9902	1.6840	0.5698
	NDUFA7	N.D.	2.7508	N.D.	1.3772
	NDUFA10	0.2821	3.3861	2.9680	0.3574
	NDUFB9	1.0159	3.7999	N.D.	0.3010
	NDUFB10	1.2863	3.5364	N.D.	0.3010
	NDUFS1	0.3133	2.6393	0.2753	0.0428
	NDUFV1	0.1862	2.6511	N.D.	0.3010
	NDUFS5	0.6779	2.7266	-0.4286	0.1035
	ATP6V1A	0.5678	1.0391	N.D.	0.5524
	ATP6V1E1	0.7282	1.1476	-0.7993	0.1219
	SDHA	1.0321	2.2107	-0.8526	0.2363
	SDHB	1.1390	2.4990	2.8721	0.2634
	UQCRB	0.9119	3.2113	N.D.	0.3010
	UQCRC1	-0.1890	2.8708	0.6729	0.3875
	UQCRC2	-0.0534	2.5270	-0.1675	0.0938
	COX5A	0.2191	2.4274	0.5794	0.0902

[1] Italicized proteins are highlighted in Figure S8.
[2] Proteins that were not detected in both samples are labeled N.D. (not detected). However, these proteins may have a p-value if the protein was detected in one sample but not the other sample, e.g. iPSC-derived neurons but not the transdifferentiated neurons. This is because a zero value would allow the calculation of a p-value even though a fold change cannot be calculated. These values were not plotted in Extended Data Figure 8.
[3] P-values were calculated using a two-tailed Welch's t-test.

Supplementary Table 6: Aged human brain tissue used in this manuscript.

	ID#	Age	Gender
Mid-Age Cohort	Case 20	38	M
	Case 26	49	M
	Case 131	56	M
Old-Age Cohort	Case 65	82	M
	Case 67	77	M
	Case 103	92	F

Supplementary Table 7: List of significantly up- or down-regulated gene ontology pathways in human brain cohorts.

	Gene Ontology Pathway	ID	# of Genes	Enrichment Score[1]	Adjusted p-value
Top 10 Pathways	Monoatomic ion transmembrane transport	0034220	415	0.510113	2.9E-17
	Oxidative phosphorylation	0006119	111	0.694098	5.46E-16
	Inorganic ion transmembrane transport	0098660	375	0.511679	9.4E-16
	Aerobic respiration	0009060	149	0.634649	1.52E-15
	Trans-synaptic signaling	0099537	428	0.486166	2.99E-15
	Cellular respiration	0045333	178	0.601564	4.91E-15
	Monoatomic cation transmembrane transport	0098655	355	0.509226	4.91E-15
	Chemical synaptic transmission	0007268	421	0.486563	4.91E-15
	Anterograde trans-synaptic signaling	0098916	421	0.486563	4.91E-15
	Synaptic signaling	0099536	445	0.474758	1E-14
RNA Pathways	RNA localization	0006403	127	-0.37692	0.004128
	Negative regulation of RNA biosynthetic process	1902679	323	-0.28716	0.007133
	Mitochondrial RNA metabolic process	0000959	31	0.606967	0.01296
	Negative regulation of transcription by RNA polymerase	0000122	222	-0.31389	0.013857
	mRNA transport	0051028	80	-0.41443	0.014455
	RNA export from nucleus	0006405	61	-0.43437	0.018037
	RNA transport	0050658	103	-0.35528	0.025208
	Establishment of RNA localization	0051236	104	-0.35184	0.029524
	RNA-templated DNA biosynthetic process	0006278	41	-0.45841	0.032459
	Negative regulation of RNA metabolic process	0051253	375	-0.25531	0.045434
	Positive regulation of RNA biosynthetic process	1902680	465	-0.23882	0.047377

[1] P-values were calculated by performing a hypergeometric test.

Supplementary Table 8: List of cell lines used in this paper.

Identifier	Age	Sex	Source	Identifier
Tdiff.1[1]	52	F	This paper	n/a
iPSC-diff.1[1]	52[2]	F	This paper	n/a
iPSC.1[1]	52[2]	F	This paper	n/a
Fibroblast.1[1]	52	F	This paper	n/a
Tdiff.2	63	M	This paper	n/a
Tdiff.3	70	F	NIA	AG07309
Tdiff.4	85	F	NIA	AG13077
Tdiff.Y	Neonatal	M	ATCC	CRL-2522

[1] Tdiff.1 is isogenic to the iPSC-diff.1 and Fibroblast.1 lines used throughout the paper.

[2] The effective DNAm age of these cell lines are -9 months-old according to the data in Figure 1D.

Supplementary Table 9: List of antibodies used for immunofluorescence in this study.

Antibody	Catalog #	Dilution
Chicken Anti-Map2[1]	Sigma #AB15452	1:500
Chicken Anti-Map2[1]	Invitrogen PA1-10005	1:250
Chicken Anti-Tubulin β3[2]	Novus #NB100-1612	1:500
Donkey Anti-Chicken IgY, Alexa Fluor 488	Thermo #A-78948	1:2000
Donkey Anti-Mouse IgG, Alexa Fluor 555	Thermo #A-31570	1:2000
Donkey Anti-Rabbit IgG, Alexa Fluor 647	Thermo #A-31573	1:2000
Mouse Anti-Caprin1	Proteintech #66352-1-Ig	1:100
Mouse Anti-FUS	Santa Cruz Biotech #sc-47711	1:200
Mouse Anti-G3BP1	Santa Cruz Biotech #sc-365338	1:1000
Mouse Anti-Synaptophysin	Sigma #55768	1:100
Rabbit Anti-AIF	Proteintech #17984-1-AP	1:100
Rabbit Anti-c-casp-3	Cell Signaling Tech #9664L	1:500
Rabbit Anti-eIF2α	Proteintech #11170-1-AP	1:200
Rabbit Anti-HSP90α	Thermo #PA3-013	1:100
Rabbit Anti-NeuN/RBFOX3	Sigma #MABN140	1:100
Rabbit Anti-PRPF8	Abcam #185547	1:100
Rabbit Anti-PSD95	Abcam #ab76115	1:100
Rabbit Anti-SNRPA	Proteintech #10212-1-AP	1:100
Rabbit Anti-SNRNP70	Thermo #PA5-115943	1:100
Rabbit Anti-SNRNP200	Abcam #AB176715	1:200
Rabbit Anti-TIA1	Proteintech #12133-2-AP	1:200
Rabbit Anti-TDP-43	Proteintech #10782-2-AP	1:200
Rabbit Anti-Tubulin β3[2]	Abcam #ab52623	1:500

[1] The Sigma antibody was used for Figures 1C. The Invitrogen antibody was used for Extended Data Figures 1D-G.

[2] The rabbit antibody was used for Extended Data Figure 1D; the chicken antibody was used for all other experiments.

Supplementary Table 10: List of antibodies used for Western blots in this study.

Antibody	Catalog #	Dilution
IRDye® 680RD Goat anti-Rabbit IgG Secondary Antibody	Li-Cor #926-68071	1:5000
Mouse Anti-GAPDH	Sigma #MAB374	1:10000
Mouse Anti-p16^{INK4A}	Novus #NBP2-37736	1:2000
Mouse Anti-Ubiquitin	Thermo #13-1600	1:500
Mouse TrueBlot: Anti-Mouse Ig HRP	Rockland #18-8817-30	1:2000
Rabbit Anti-Caprin1	Bethyl #A303-882A	1:1000
Rabbit Anti-eIF2α	Cell Signaling Tech #9722S	1:1000
Rabbit Anti-Phospho-eIF2α	Cell Signaling Tech #9721S	1:500
Rabbit Anti-G3BP1	MBLI #RN048PW	1:1000
Rabbit Anti-HSP90α	Thermo #PA3-013	1:1000
Rabbit Anti-TDP-43	Proteintech #10782-2-AP	1:1000
Rabbit TrueBlot: Anti-Rabbit IgG HRP	Rockland #18-8816-31	1:2000

Supplementary Table 11: List of antibodies used for immunoprecipitation experiments in this study.

Antibody	Catalog #	Dilution
Rabbit Anti-Caprin1	Bethyl #A303-882A	10 μg (eCLIP)
Rabbit Anti-G3BP1	MBLI #RN048PW	10 μg (eCLIP); 6 μg (AP-MS)
Rabbit Anti-HSP90α	Thermo #PA3-013	6 μg (AP-MS)
Rabbit Anti-TDP-43	Bethyl #A303-223A	5 μg (eCLIP); 3 μg (AP-MS)
