# Supplementary figures and images for: Neuronal aging causes mislocalization of splicing proteins and unchecked cellular stress
Source: Nat Neurosci. 2025 Jun 2;28(6):1174–84. doi: 10.1038/s41593-025-01952-z (PMC12148940; doi:10.1038/s41593-025-01952-z)

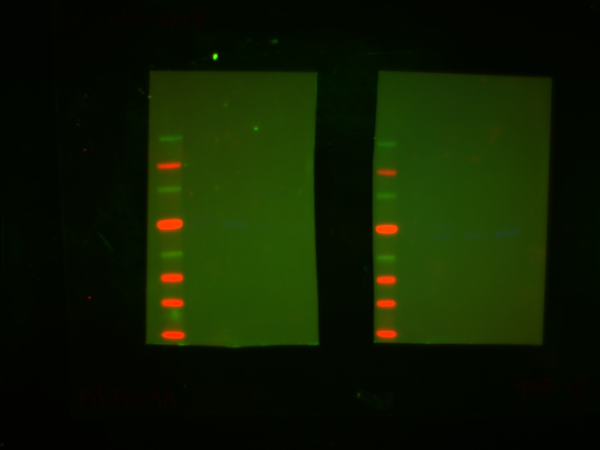

Supplement: Supplementary file 3 — Unprocessed p16INK4A western blot. [file 41593_2025_1952_MOESM3_ESM.tif]

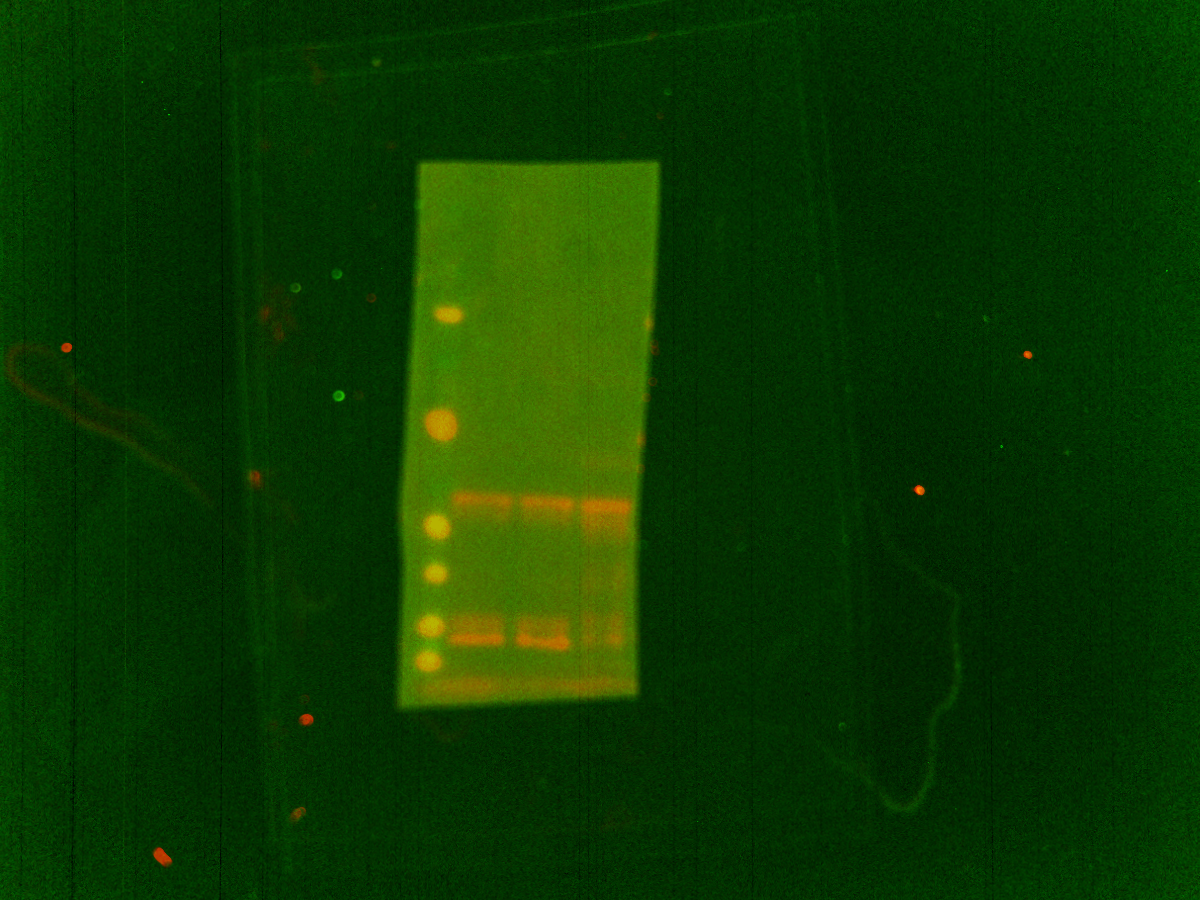

Supplement: Supplementary file 4 — Unprocessed eIF2α western blot. [file 41593_2025_1952_MOESM4_ESM.tif]

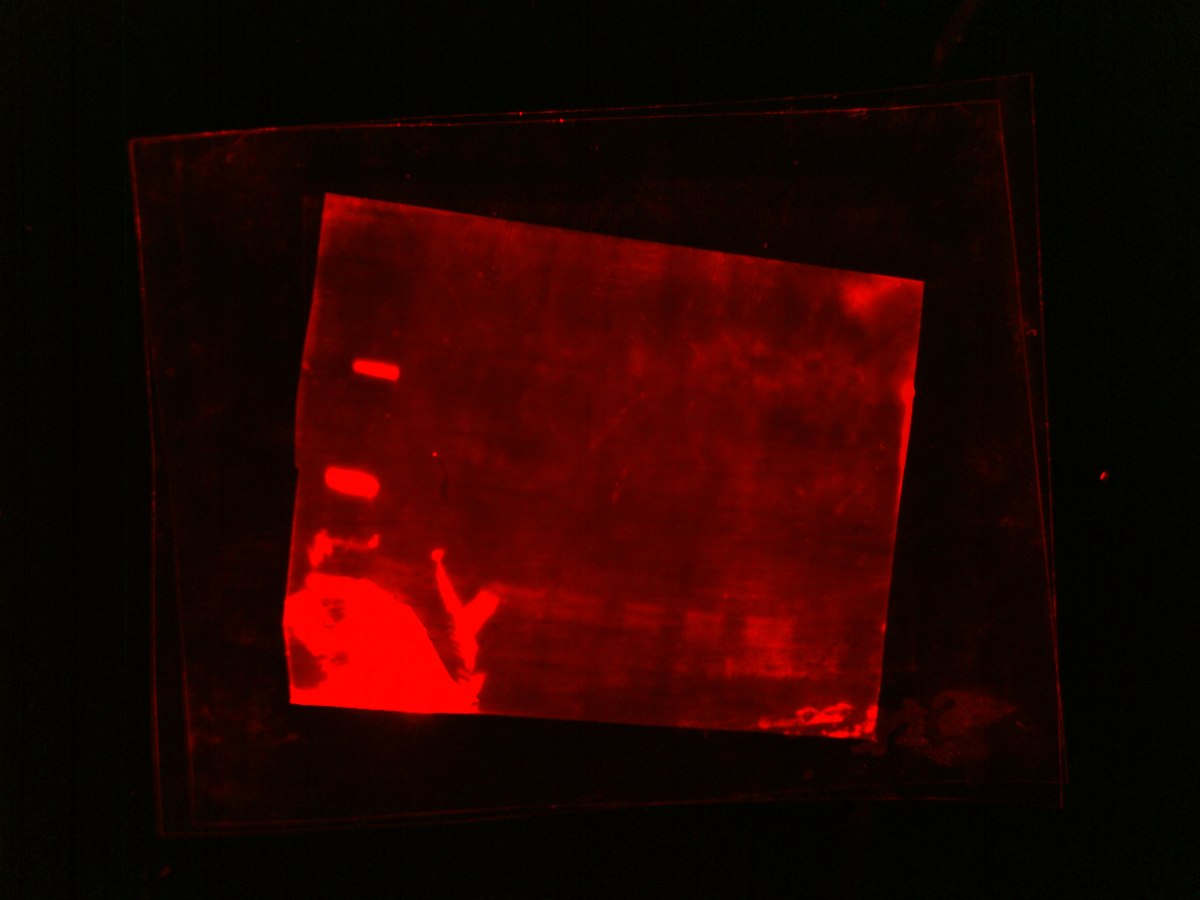

Supplement: Supplementary file 5 — Unprocessed eIF2α western blot. [file 41593_2025_1952_MOESM5_ESM.tif]
